# Regression calibration utilizing biomarkers developed from high-dimensional metabolites

**DOI:** 10.3389/fnut.2023.1215768

**Published:** 2023-08-02

**Authors:** Yiwen Zhang, Ran Dai, Ying Huang, Ross L. Prentice, Cheng Zheng

**Affiliations:** ^1^Zilber School of Public Health, University of Wisconsin-Milwaukee, Milwaukee, WI, United States; ^2^Department of Biostatistics, University of Nebraska Medical Center, Omaha, NE, United States; ^3^Public Health Science Division, Fred Hutchinson Cancer Center, Seattle, WA, United States

**Keywords:** measurement error, regression calibration, feeding study, biomarker, high-dimensional data

## Abstract

Addressing systematic measurement errors in self-reported data is a critical challenge in association studies of dietary intake and chronic disease risk. The regression calibration method has been utilized for error correction when an objectively measured biomarker is available; however, biomarkers for only a few dietary components have been developed. This paper proposes to use high-dimensional objective measurements to construct biomarkers for many more dietary components and to estimate the diet disease associations. It also discusses the challenges in variance estimation in high-dimensional regression methods and presents a variety of techniques to address this issue, including cross-validation, degrees-of-freedom corrected estimators, and refitted cross-validation (RCV). Extensive simulation is performed to study the finite sample performance of the proposed estimators. The proposed method is applied to the Women's Health Initiative cohort data to examine the associations between the sodium/potassium intake ratio and the total cardiovascular disease.

## 1. Introduction

The field of nutritional epidemiology plays a crucial role in understanding the impact of dietary patterns on human health. The ongoing exploration of associations between dietary components and chronic disease risks continually uncovers valuable insights. For instance, the well-established link between obesity and cancer risk (1) serves as a testament to the significance of this research. In order to effectively prevent and control chronic diseases, it is imperative to acquire detailed information on how key energy balance factors associate with the risks of major chronic illnesses [World Cancer Research Fund/American Institute for Cancer Research (2)]. Investigating the complex working mechanisms of these energy balance factors necessitates a comprehensive examination of the connections between multiple dietary components and disease risks. Establishing such associations, however, is far from simple. A major challenge stems from biases in dietary assessment, which are notoriously difficult to address (3). Strong evidence (4) suggests that the misreporting of dietary energy intake is associated with individual characteristics, such as body mass index (BMI). These systematic measurement errors result in estimation biases that cannot be automatically rectified (5). Moreover, correcting measurement errors becomes increasingly challenging when attempting to model dietary components jointly in the context of their relationships with chronic diseases.

Correcting measurement errors has been an important subject in statistical methodology development, greatly influencing nutritional studies (6). Various strategies have been developed to address these errors (7–15). One notable method, regression calibration, is particularly useful for handling covariate-dependent measurement errors and offers ease of implementation (16). Studies within the Women's Health Initiative (WHI) have demonstrated the effectiveness of joint regression calibration approaches in addressing measurement errors when objective biomarkers are available for all modeled dietary intakes (4, 17–19). These biomarkers inform calibration equations for self-reported measurements of exposure variables, which then provide calibrated intake estimates to better assess associations between dietary exposures and disease risks.

There is a significant research gap in generating reliable calibrated estimates for numerous nutritional and physical activity variables using single objective measurements. Consequently, regression models with multiple predictors have been developed from feeding studies to obtain calibrated estimates (4, 20). For instance, in the WHI Nutrition and Physical Activity Assessment Study (NPAAS), a regression-based biomarker has been established for a single dietary component or energy balance factors (21). To address the systematic measurement errors in self-reported food frequency questionnaire (FFQ) data from a large cohort, blood and urine measurements were collected for a subgroup, while a feeding study (NPAAS-FS) was conducted on another smaller subgroup where both blood and urine measurements and assessed dietary intake information were collected. This novel feeding study design aimed to improve the accuracy of capturing measurement errors in the FFQ (21). However, there are some challenges in building the regression calibration method, as the classical measurement error assumption will be violated by the feeding study-based biomarker development procedure, which regresses the consumed nutrient on blood and urine measurements and personal characteristics. This issue arises because the residual of the regression model is independent of the predicted value instead of the actual one. Ignoring this violation results in biased estimates of the calibrated dietary intake and the diet-disease association due to Berkson-type errors (22). When developing biomarkers for objectively measured variables with low dimensions, new calibration methods have been developed to account for Berkson-type errors in association studies of univariate nutritional variables (20). Zhang et al. (23) have extended this approach to multivariate nutritional variables, providing consistent estimators for disease associations of a single dietary component and valid confidence intervals for disease association parameters under rare disease settings. Nevertheless, for some macronutrient intakes, suitable biomarkers cannot be developed from low-dimensional measurements. High-dimensional metabolites offer an opportunity to establish valid biomarkers, but it remains an open question on how to obtain valid inferences for such biomarkers.

In this paper, we concentrate on high-dimensional objective measurements for a univariate exposure of interest, where the sample size is smaller than the dimension of the variables, in constructing a biomarker model. High-dimensional variable selection constitutes a significant portion of the rapidly advancing statistical frontiers today. Over the past few decades, numerous studies have been dedicated to understanding the performance of various variable selection techniques. Frank and Friedman (24) first proposed a technique called bridge regression. Breiman (25) introduced the nonnegative garrote for shrinkage estimation and variable selection. Lasso, an *l*-1 regularized least squares method, was studied and introduced by (26) for variable selection. Nonconcave penalized likelihood estimators, such as smoothly clipped absolute deviation (SCAD), were proposed by (27) and (28). Efron et al. (29) presented the least angle regression for variable selection and introduced the LARS algorithm. Zou and Li (30) proposed one-step sparse estimates for nonconcave penalized likelihood models and introduced the local linear approximation algorithm for optimizing non-concave penalized likelihoods.

Building a biomarker model with high-dimensional sparse data requires predictive performance that can effectively address the challenges associated with such data. One issue that arises when working with high-dimensional models is the collinearity among covariates, which can result in spurious correlations between variables (31). Numerous researchers have explored penalized regression techniques, such as Lasso and SCAD, to handle high-dimensional sparse data. Alternatively, variable selection can also be done by ranking predictive powers using random forest (RF) (32). Variance estimation in high dimensional models presents its own challenges, due to factors such as collinearity among covariates and the presence of spurious correlations. A variety of techniques have been proposed to address the issue of variance estimation in high-dimensional regression methods. Cross-validation (CV), a popular resampling technique, has been widely applied to assess the performance of different models and obtain unbiased variance estimates (33). The bootstrap, another resampling method, has been employed to estimate the variability of regression parameters (34). The degrees-of-freedom corrected estimators, such as the generalized degrees of freedom and the effective degrees of freedom, provide better error variance estimates by accounting for the complexity of the models (35, 36). The refitted cross-validation (RCV) method is a modification of the standard cross-validation procedure that improves the estimation of error variance in high-dimensional regression (37).

The remaining of the paper is organized as follows. In Section 2, we introduce the framework of the present study and the notation. In Section 3, we introduce different methods and detail variance estimation procedures. In Section 4, we conduct extensive simulations to evaluate the finite sample performance of our proposed estimators. In Section 5, we apply our method to the WHI data to estimate the effect of macronutrient intakes on the risk of various chronic diseases. Finally, in Section 6, we present our conclusions and discussions.

## 2. Framework and notation

We aim to investigate the correlation between a particular type of dietary intake *Z* ∈ ℝ [such as the (log-transformed) ratio of dietary sodium to potassium] and the timeframe, *T*, to the emergence of a specific chronic illness. Nevertheless, rather than directly observing *Z*, we only gather information on self-reported dietary intake *Q* ∈ ℝ, which may deviate from *Z* depending on individual characteristics:


(1) $$\begin{array}{l} Q=\left(1,Z,{\;V\;}^{⊤}\right)\;\text{a }+\epsilon_{q}, \end{array}$$

Here, ***a*** ∈ ℝ^(2+*q*)^ is an unidentified parameter vector, and ϵ_*q*_ is a random error with a mean of 0 that is independent of *Z* and ***V***. We also take into account potential confounding factors, referred to as personal characteristics ***V*** ∈ ℝ^*q*^ where *q* is the number of covariates. To model the hazard of the response, we employ a Cox model:


(2) $$\begin{array}{l} \lambda \left(t|Z,\;\text{V },Q\right)=\lambda \left(t|Z,\;\text{V }\right)=\lambda_{0}\left(t\right)\exp\left(\left(Z,{\;\text{V }}^{⊤}\right)\;\theta \;\right), \end{array}$$

where $\;\theta \;={\left(\theta_{z},{\;\theta \;}_{v}^{⊤}\right)}^{⊤}\in {\mathbb{R}}^{\left(1+q\right)}$, θ_*z*_ is the parameter we are interested in, and λ_0_(*t*) represents a “baseline” hazard function.

In the NPAAS feeding study (NPAAS-FS), we furnish participants' meals with standardized food, which closely mimicking their regular diet, has well-documented nutrient content (21). The true unobservable dietary intake within the 2-week feeding period is denoted as


(3) $$\begin{array}{l} X=Z+\epsilon_{x},\text{ where }\epsilon_{x}\sim N\left(0,\sigma_{x}^{2}\right). \end{array}$$

In our current model, we assume that ϵ_*x*_ is independent of *Z* and ***V***. However, condition (3) could be considered somewhat restrictive, given that the design of the feeding study is based on reported long-term dietary intake and not the actual diet. To address this, we have modified this assumption such that the true short-term unobserved diet *X* does not necessarily need to be centered around *Z*. Additional specifics can be found in Section 3. One intricate issue related to the feeding study is the measurement errors arising from food packaging. For example, a pack of chips labeled as 100 calories might in reality contain 101 calories. Consequently, the observed short-term dietary intake $\tilde{X}$ during the feeding study can be expressed as $\tilde{X}=X+{\tilde{\epsilon }}_{x}$, where ${\tilde{\epsilon }}_{x}\sim N\left(0,{\tilde{\sigma }}_{x}^{2}\right)$ is independent of ϵ_*x*_, *Z*, and ***V***.

The study is organized into three stages: the feeding study (Sample 1) for biomarker development, the biomarker sub-study (Sample 2) for calibration equation development, and the association study (Sample 3) using the complete cohort to establish the disease association.

When self-reported intake *Q* data from feeding study samples is available, the bias of self-reported dietary intake can be directly calibrated (refer to Section 3.4). However, self-reported dietary intake *Q* is usually not available concurrently in Sample 1. To acquire that data, a long-term feeding study would be necessary, wherein participants report their dietary intake provided over preceding months (e.g., 3 months). Furthermore, the *Q* value obtained just prior to the feeding period in NPAAS-FS is not collected at the same time as biomarker *W*, and it might be inappropriately highly correlated with $\tilde{X}$.

As an alternative, we could employ a high-dimensional biomarker ***W*** ∈ ℝ^*p*^, comprised of *p* blood and urine measurements obtained objectively, as a bridge between $\tilde{X}$ from the feeding study sample and *Q* from a separate, larger sample. We assume that the blood and urine measurements *W* are influenced by the short-term diet *X*, whereas the self-reported questionnaire data are directly impacted by the long-term diet *Z*. We assume that ***W*** is possibly high-dimensional and follows a parametric model:


$$\begin{array}{l} \text{W }={\left[\left(1,X,{\;\text{V }}^{⊤}\right)\;\text{B }\right]}^{⊤}+\epsilon_{w}, \end{array}$$

where ***B*** ∈ ℝ^(2+*q*) × *p*^ is a matrix of unknown parameters and $\epsilon_{w}\sim N\left(0,\sigma_{w}^{2}I_{p}\right)$ is independent of $\epsilon_{x},{\tilde{\epsilon }}_{x},\epsilon_{q},Z,\;V\;\text{and}\;B\;$.

In practical terms, our best option is to utilize the baseline *Q* gathered at a separate time (for instance, at baseline for Sample 3) for Sample 1. This baseline *Q* has been effectively used in studies concerning various dietary components [e.g., protein and carbohydrate; (22)]. However, a time gap exists between the data collection for this baseline *Q* and the timing of $\left(\tilde{X},\;V\;,\;W\;,Z\right)$ measurements in Sample 1. Consequently, there's a concern that the conditional distribution $\left(Q|\tilde{X},\;V\;,\;W\;,Z\right)$ in Sample 1 may differ from Samples 2 and 3 for specific dietary components. Even when *Q* is available, the feeding study's sample size is usually restricted, which could lead to less than optimal efficiency for disease association estimates. In such instances, we consider *Q* as unavailable in Sample 1 and use ***W*** to predict $\tilde{X}$.

The process of estimating the association between *Z* and *T* is divided into three stages, each utilizing distinct, non-overlapping samples derived from the same fundamental population: 1. the biomarker creation phase, 2. the calibration phase, and 3. the phase assessing the association. Each stage employs a different sample. The size of the sample used in stage *k* is denoted as *n*_*k*_. In Stage 1, there are *n*_1_ samples, and for each individual *i*, we have access to data $\left({\tilde{X}}_{i},{\;W\;}_{i}^{⊤},{\;V\;}_{i}^{⊤}\right)$ and possibly *Q*_*i*_; in Stage 2, *n*_2_ samples are available, and for each individual *i*, we have $\left(Q_{i},{\;W\;}_{i}^{⊤},{\;V\;}_{i}^{⊤}\right)$; in Stage 3, there are *n*_3_ samples, and for each individual *i*, we have $\left(Q_{i},{\;V\;}_{i}^{⊤}\right)$ and the composite outcome $\left[T_{i}^{*}=T_{i}\wedge C_{i},\Delta_{i}=I\left(T_{i}\leq C_{i}\right)\right]$, where *T*_*i*_ is the time of disease occurrence, and *C*_*i*_ is a potential censoring time. Conventionally, *T*_*i*_ and *C*_*i*_ are assumed to be independent given $\left(Q_{i},{\;V\;}_{i}^{⊤}\right)$.

During the first stage, we utilize data from the biomarker creation phase to develop the biomarker. This model can be constructed by regressing the observed short-term dietary intakes $\tilde{X}$ on one of the following:

(i) blood/urine measurements ***W*** and personal characteristics ***V***;
(ii) blood/urine measurements ***W***, self-reported dietary intake *Q*, and personal characteristics ***V***;
(iii) self-reported dietary intake *Q* and personal characteristics ***V***.

As earlier indicated, self-reported dietary intake *Q* may be deemed unavailable during Stage 1. If that's the case, we treat *Q* as unavailable and opt for choice (i) in Stage 1. When *Q* is accessible in Stage 1, choice (ii) might enhance the estimation of $\tilde{X}$. If the biomarker *W* is not available, option (iii) directly models $\tilde{X}$ based on *Q* and *V*, but the effectiveness might be hampered by the limited sample size *n*_1_. In Stage 2, a calibration equation is developed using self-reported log-transformed dietary intake *Q* and personal characteristics ***V*** to predict actual intake *X* if options (i) or (ii) are implemented in Stage 1. If option (iii) is chosen, Stage 2 is bypassed, as the equation is already established in Stage 1. However, option (i) introduces Berkson-type error, which impacts regression calibration, thus necessitating new methodologies to address this characteristic. In Stage 3, we calibrate the self-reported dietary intake utilizing the Stage 2 calibration equation, conduct disease association analyses with the available data on *Q*, ***V***, and the composite survival outcome (T^*^, Δ).

In summary, the high-dimensional regression calibration procedure has three stages: biomarker construction, calibration, and estimation. In Stage 1, the relationship between the true dietary intake ***X*** and high-dimensional biomarker ***W*** is established. If self-reported dietary intakes ***Q*** are not available, option (i) can be used. If ***Q*** is available in Stage 1, whether or not ***W*** is also available, relationships between ***X*** and ***Q*** can be directly established with option (iii). If both ***W*** and ***Q*** are available for Stage 1, one of the options from (i), (ii), and (iii) can be used. As discussed, (i) might lead to Berkson type error (38) and (iii) might have low efficiency. For Stage 2, we developed bias correction methods to account for the bias introduced by the Berkson type error. For Stage 3, we can use a multivariate approach to jointly study the associations between multiple dietary components and the disease risks.

## 3. Methods

we first consider the case where ${\tilde{\sigma }}_{x}$ is known. We propose methods to estimate ${\tilde{\sigma }}_{x}$ in the discussion section. In the real data analysis where ${\tilde{\sigma }}_{x}$ is not available, we vary this parameter to perform sensitivity analysis.

With high-dimensional data on urine measurements (***W***), we first need to obtain estimated coefficients among *n*_1_ subjects in the biomarker discovery sample of the observed short-term dietary intake $\tilde{X}$ on high-dimensional blood and urine measurements (***W***) as well as subject characteristics (***V***). Three different approaches including Lasso, SCAD, and RF are used to conduct variable selection in high-dimensional statistical inference. We will describe each approach explicitly for every method in the following subsections.

### 3.1. Method 1: the naïve three-step approach with multiple exposure

In the first step, we need to fit a linear regression of $\tilde{X}$ on ***W*** and ***V***:


$$\begin{array}{l} 𝔼\left(\tilde{X}|\;\text{W },\;\text{V }\right)=\left(1,\;\text{W },\;\text{V }\right)\beta_{1}. \end{array}$$

With Lasso approach, the coefficients, ${\hat{\beta }}_{1}$, minimize the penalized least squares (*PL*_*Lasso*_) as below:


$$\begin{array}{l} PL_{Lasso}=\overset{n}{\underset{i=1}{\sum }}{\left({\tilde{X}}_{i}-\left(1,{\;\text{W }}_{i}^{⊤},{\;\text{V }}_{i}^{⊤}\right)\beta_{1}\right)}^{2}+\lambda \overset{p}{\underset{j=1}{\sum }}|\beta_{1j}|. \end{array}$$

Lasso performs variable selection by shrinking coefficient estimates toward zero leading to a sparse model. The tuning parameter λ is selected through cross-validation.

With the SCAD approach, a nonconvex penalty is given by:


$$\begin{array}{l} PL_{SCAD}\left(\beta_{1j}\right)=\{\begin{array}{c} \lambda |\beta_{1}|\;if|\beta_{1j}|<\lambda \\ 2a\lambda |\beta_{1}{|}^{2}-2a\lambda |\beta_{1}|\;if\lambda <|\beta_{1j}|<a\lambda \\ \left(a+1\right)\lambda^{2}/2\;if|\beta_{1j}|>a\lambda \end{array} \end{array}$$

The first derivatives of *PL*_*SCAD*_(β_1*j*_) is continuous and is given by


$$\begin{array}{l} PL_{SCAD}{\left(\beta_{1}\right)}^{\prime }=\lambda \left\{I\left(\beta_{1}<\lambda \right)+\frac{{\left(a\lambda -\beta_{1}\right)}_{+}}{\left(a-1\right)\lambda }I\left(\beta_{1}>\lambda \right)\right\} \end{array}$$

for some *a* > 2 and β_1_ > 0. Similar to Lasso, λ in SCAD is selected through cross-validation based on the smallest mean square error (MSE) whereas *a* is set to be 3.7 based on simulation results and Bayesian statistical point of view from (27).

Other than penalized regression as we described above, RF is another choice for variable selection. The basic concept is to grow regression trees in the general form below:


$$\begin{array}{l} 𝔼\left[\tilde{X}|\;\text{W },\;\text{V }\right]=\overset{M}{\underset{m=1}{\sum }}c_{m}1_{\left(\;\text{W },\;\text{V }\right)\in R_{m}} \end{array}$$

where *R*_1_, …, *R*_*M*_ denotes a partition of feature space. Then we can repeat this procedure to build the RF by considering the approximate square root of the total number of predictors each time. The advantage of RF is we can see the contribution of each variable to the regression tree and their relative importance.

For each method, we did direct selection and post selection. For direct selection, we applied an estimated model from each approach to predict the long-term dietary intake straightly. For post selection, we performed linear regression afterward with selected variables (Ŝ) from each approach. Specifically, for Lasso and SCAD, we have:


$$\begin{array}{l} {\hat{\beta }}_{1}=argmin\left\{\overset{n}{\underset{i=1}{\sum }}{\left({\tilde{X}}_{i}-\left(1,{\;\text{W }}_{i}^{⊤},{\;\text{V }}_{i}^{⊤}\right)\beta_{1}\right)}_{2}^{2}\right\}. \end{array}$$

$$\begin{array}{l} \beta_{1}\in {\mathbb{R}}^{P}\;and\;{\hat{\beta }}_{1j}=0\;\forall \;j\notin Ŝ \end{array}$$

For RF, the 10 most important variables are considered as the final selected variables. For both direct and post selection, we considered two ways to deal with ***W*** and ***V***; one is to consider both ***W*** and ***V*** in the approach of variable selection while the other is to consider only ***W***. To be more specific, in Lasso and SCAD, the penalization will be applied to (***W***, ***V***) or to only ***W***, respectively. In RF, the decision trees will be built by considering (***W***, ***V***) or only ***W***, respectively.

With estimated ${\hat{\beta }}_{1}$ we had in the prior step, we can then compute ${\hat{X}}_{1i}=\left(1,{\;W\;}_{i}^{⊤},{\;V\;}_{i}^{⊤}\right){\hat{\beta }}_{1}$ to predict the long-term dietary intake (*Z*) among the *n*_2_ calibration samples and run a regression of ${\hat{X}}_{1}$ on self-reported food frequency questionnaire data (*Q*) and ***V*** to build calibration equation using the *n*_2_ calibration samples to estimate the parameter


$$\begin{array}{l} {\hat{\gamma }}_{1}={\left\{\overset{n_{1}+n_{2}}{\underset{i=n_{1}+1}{\sum }}{\left(1,Q_{i},{\;\text{V }}_{i}^{⊤}\right)}^{⊤}\left(1,Q_{i},{\;\text{V }}_{i}^{⊤}\right)\right\}}^{-1}\left\{\overset{n_{1}+n_{2}}{\underset{i=n_{1}+1}{\sum }}{\left(1,Q_{i},{\;\text{V }}_{i}^{⊤}\right)}^{⊤}{\hat{X}}_{1i}\right\}. \end{array}$$

Using the Stage 3 sample, we then estimate *Z* as ${\hat{Z}}_{1i}=\left(1,Q_{i},{\;V\;}_{i}^{⊤}\right){\hat{\gamma }}_{1}$ for *i* = *n*_1_ + *n*_2_ + 1, ⋯ , *n*_1_ + *n*_2_ + *n*_3_. Finally, we estimate the association between *Z* and the time-to-event endpoint (T^*^, Δ) by solving the score equation for Cox model:


$$\begin{array}{l} 0=\sum_{i=n_{1}+n_{2}+1}^{n_{1}+n_{2}+n_{3}}\int_{0}^{\tau }[\left(\begin{array}{c} {\hat{Z}}_{1i}\\ {\text{V}}_{i} \end{array}\right)\\ \;-\sum_{j}\frac{Y_{j}(t)\exp\left\{({\hat{Z}}_{1j},{\text{V}}_{j}^{⊤})\theta \right\}}{\sum_{k}Y_{k}(t)\exp\left\{({\hat{Z}}_{1k},{\text{V}}_{k}^{⊤})\theta \right\}}\left(\begin{array}{c} {\hat{Z}}_{1j}\\ {\text{V}}_{j} \end{array}\right)]dN_{i}(t). \end{array}$$

where τ is a pre-specified large number and we assume *P*(*C* > τ) > 0, $N_{i}\left(t\right)=I\left(\Delta_{i}=1,T_{i}^{*}\leq t\right)$ and $Y_{i}\left(t\right)=I\left(T_{i}^{*}\geq t\right)$. In application, τ is typically defined as the largest follow-up time in the Stage 3 sample.

### 3.2. Method 2: three-step with bias correction

As shown in (20), for low-dimension setting, Method 1 will lead to a bias factor in Ẑ_1_ when using ${\hat{X}}_{1}$ and a bias-corrected estimator has been proposed. so for this high dimensional setting, we propose a similar bias-corrected estimator ${\hat{X}}_{2}={\hat{X}}_{1}{\hat{BF}}^{-1}$ where


$$\begin{array}{l} \hat{BF}={\hat{R}}_{1|\;\text{V }}^{2}=1-\frac{\hat{Var}\left(\tilde{X}|\;\text{V },\;\text{W }\right)-{\tilde{\sigma }}_{x}^{2}}{\hat{Var}\left(\tilde{X}|\;\text{V }\right)-{\tilde{\sigma }}_{x}^{2}} \end{array}$$

is an estimated version of the bias factor.

For direct selection, we used *K*-fold cross-validated errors to compute the $\hat{Var}\left(\tilde{X}|{\;W\;}_{s},{\;V\;}_{s}\right)$ in penalized regression and RF to obtain $\hat{BF}$. Denote the predicted value for the *k* − *th* fold when using regression parameters from the other *K* − 1 training datasets as ${\hat{\tilde{X}}}_{1k}$ when using (***W***,***V***) as predictors and as ${\hat{\tilde{X}}}_{2k}$ when using ***V*** as predictors, then we have


$$\begin{array}{l} {\hat{\tilde{X}}}_{1}=\left({\hat{\tilde{X}}}_{11},{\hat{\tilde{X}}}_{12},\cdots \;,{\hat{\tilde{X}}}_{1k}\right), \end{array}$$

$$\begin{array}{l} {\hat{\tilde{X}}}_{2}=\left({\hat{\tilde{X}}}_{21},{\hat{\tilde{X}}}_{22},\cdots \;,{\hat{\tilde{X}}}_{2k}\right). \end{array}$$

With ${\hat{\tilde{X}}}_{1}$ and ${\hat{\tilde{X}}}_{2}$, $\hat{BF}$ can be calculated as


$$\begin{array}{l} \hat{BF}=1-\frac{n^{-1}\sum_{i=1}^{n}{\left({\tilde{X}}_{i}-{\hat{\tilde{X}}}_{1i}\right)}^{2}-{\tilde{\sigma }}_{x}^{2}}{n^{-1}\sum_{i=1}^{n}{\left({\tilde{X}}_{i}-{\hat{\tilde{X}}}_{2i}\right)}^{2}-{\tilde{\sigma }}_{x}^{2}}. \end{array}$$

For post selection, we first obtain the selected variables from the methods Lasso, SCAD, or RF. Afterward, we estimate the coefficients by refitting a linear regression. To facilitate interpretation, we consider both ***W*** and ***V*** in variable selection for the remainder of this subsection. Consequently, we have:


$$\begin{array}{l} \tilde{X}\sim \left({\;\text{W }}_{s},{\;\text{V }}_{s}\right)\beta_{WV}^{PS}. \end{array}$$

Subsequently, we can fit a low-dimensional model as below:


$$\begin{array}{l} \tilde{X}\sim \;\text{V }\beta_{V}. \end{array}$$

Here, ***W***_*s*_ and ***V***_*s*_ denote the selected ***W*** and ***V*** variables, while $\beta_{WV}^{PS}$ and β_*V*_ represent the corresponding coefficients in the aforementioned equations. From there, *BF* can be estimated as:


$$\begin{array}{l} \hat{BF}=1-\frac{\hat{Var}\left(\tilde{X}|{\;\text{W }}_{s},{\;\text{V }}_{s}\right)-{\tilde{\sigma }}_{x}^{2}}{\hat{Var}\left(\tilde{X}|\;\text{V }\right)-{\tilde{\sigma }}_{x}^{2}}, \end{array}$$

where


$$\begin{array}{l} \hat{Var}\left(\tilde{X}|{\;\text{W }}_{s},{\;\text{V }}_{s}\right)=n^{-1}\overset{n_{1}}{\underset{i=1}{\sum }}{\left({\tilde{X}}_{i}-\left(W_{si},V_{si}\right){\hat{\beta }}_{WV}^{PS}\right)}^{2},\text{and} \end{array}$$

$$\begin{array}{l} \hat{Var}\left(\tilde{X}|\;\text{V }\right)=n^{-1}\overset{n_{1}}{\underset{i=1}{\sum }}{\left({\tilde{X}}_{i}-V_{i}{\hat{\beta }}_{V}\right)}^{2}. \end{array}$$

As demonstrated above, obtaining a precise estimation of $\hat{Var}\left(\tilde{X}|{\;W\;}_{s},{\;V\;}_{s}\right)$ is crucial for a reliable estimation of *BF*. Chatterjee and Jafarov (39) revealed that the estimator $\hat{Var}\left(\tilde{X}|{\;W\;}_{s},{\;V\;}_{s}\right)$, as mentioned earlier, leads to a downward bias when using Lasso. Therefore, we decide to compute and compare three different types of $\hat{Var}\left(\tilde{X}|{\;W\;}_{s},{\;V\;}_{s}\right)$ in our study involving post selection. We will provide a description of each type below.

(i) K-fold cross validation We fit penalized regression or RF with the cross-validated training dataset and get predicted $\tilde{X}$ with selected (***W***, ***V***) for each fold. Denote the selected subset as *S*_*k*_ for each training set ${\tilde{X}}_{-k}$. Denote ***W***_*S*_*k*__ as selected ***W*** and ***V***_*S*_*k*__ as selected ***V*** in the (K-1) training dataset for each fold, then we can fit a linear regression of ${\tilde{X}}_{k}$ on ***W***_*S*_*k*__, ***V***_*S*_*k*__ and the predicted value is denoted as ${\hat{\tilde{X}}}_{1k}$. Also, we can fit a linear regression of ${\tilde{X}}_{k}$ on ***V***_*k*_ and the predicted value is denoted as ${\hat{\tilde{X}}}_{2k}$. After doing this for all *K* folds, we get the estimated values of $\tilde{X}$ for the whole sample 1, that is,
  $$\begin{array}{l} {\hat{\tilde{X}}}_{1}=\left({\hat{\tilde{X}}}_{11},{\hat{\tilde{X}}}_{12},\cdots \;,{\hat{\tilde{X}}}_{1k}\right), \end{array}$$
  $$\begin{array}{l} {\hat{\tilde{X}}}_{2}=\left({\hat{\tilde{X}}}_{21},{\hat{\tilde{X}}}_{22},\cdots \;,{\hat{\tilde{X}}}_{2k}\right). \end{array}$$
  With ${\hat{\tilde{X}}}_{1}$ and ${\hat{\tilde{X}}}_{2}$, $\hat{BF}$ can be calculated as
  $$\begin{array}{l} \hat{BF}=1-\frac{n^{-1}\sum_{i=1}^{n}{\left({\tilde{X}}_{i}-{\hat{\tilde{X}}}_{1i}\right)}^{2}-{\tilde{\sigma }}_{x}^{2}}{n^{-1}\sum_{i=1}^{n}{\left({\tilde{X}}_{i}-{\hat{\tilde{X}}}_{2i}\right)}^{2}-{\tilde{\sigma }}_{x}^{2}}. \end{array}$$
(ii) Modified variance estimator When performing penalized regression for variable selection, the choice of the regularization parameter λ is crucial for obtaining an accurate finite sample estimator. The value of λ influences both the number of variables selected and the extent to which their estimated coefficients are shrunk toward zero. If λ is set too large, not all signal variables will be selected, resulting in rapidly degrading performance (mainly characterized by a significant upward bias) as the true β becomes less sparse with a larger signal per element. Conversely, if λ is set too small, many noise variables will be selected, which allows spurious correlations to decrease our variance estimate, leading to considerable downward bias. Based on the simulation result in (40), there is a balance to be maintained when selecting the appropriate λ:
  $$\begin{array}{l} \hat{Var^{*}}\left(\tilde{X}|{\;\text{W }}_{s},{\;\text{V }}_{s}\right)={\left(n-{ŝ}_{\lambda }\right)}^{-1}\underset{i}{\sum }{\left({\tilde{X}}_{i}-\left({\;\text{W }}_{si},{\;\text{V }}_{si}\right){\hat{\beta }}_{WV}^{PS}\right)}^{2} \end{array}$$
  where ŝ_λ_ is the number of nonzero elements in $\hat{b}$ at the regulation parameter λ selected with K-fold (usually 5–10) cross-validation. Then we have:
  $$\begin{array}{l} \hat{BF}=1-\frac{\hat{Var^{*}}\left(\tilde{X}|{\;\text{W }}_{s},{\;\text{V }}_{s}\right)-{\tilde{\sigma }}_{x}^{2}}{\hat{Var}\left(\tilde{X}|\;\text{V }\right)-{\tilde{\sigma }}_{x}^{2}}. \end{array}$$
(iii) Refitted cross-validation estimator (RCV) This estimator is derived from the RCV procedure proposed by (37). We first split the dataset into two roughly equal parts: $\left[{\tilde{X}}^{\left(1\right)},{\;W\;}^{\left(1\right)},{\;V\;}^{\left(1\right)}\right]$ and $\left[{\tilde{X}}^{\left(2\right)},{\;W\;}^{\left(2\right)},{\;V\;}^{\left(2\right)}\right]$. We then perform penalized regression and RF on the first part. For penalized regression, we fit Lasso or SCAD on ***W*** and ***V*** with cross-validated $\hat{\lambda }$ to obtain the non-zero estimated coefficients for ***W*** and ***V***. In the case of RF, we select the 10 most important variables based on the residual sum of squares (RSS). We then refit the model with the selected ***W*** and ***V*** to obtain the post selected estimators of their coefficients, denoted as ${\hat{\beta }}^{PS\left(1\right)}WV$. Subsequently, using the selected ***W*** and ***V*** in ***W***^(2)^ and ***V***^(2)^, we can compute the following variance estimate on the second part.
  $$\begin{array}{l} \hat{Var_{1}^{**}}\left(\tilde{X}|{\;\text{W }}_{s},{\;\text{V }}_{s}\right)={\left(n-{ŝ}^{\left(1\right)}\right)}^{-1}\underset{i}{\sum }{\left(X_{i}^{*\left(2\right)}-\left({\;\text{W }}_{si}^{\left(2\right)},{\;\text{V }}_{si}^{\left(2\right)}\right){\hat{\beta }}_{WV}^{PS\left(1\right)}\right)}^{2}, \end{array}$$
  where ŝ^(1)^ is the number of selected variables in the first part. Repeating the mirror image procedure on $\left[{\tilde{X}}^{\left(2\right)},{\;W\;}_{s}^{\left(2\right)},{\;V\;}_{s}^{\left(2\right)}\right]$, we can obtain ${\hat{\lambda }}_{2}$, selected W obtained from Lasso in the second part and ${\hat{Var}}_{2}\left(\tilde{X}|\;W\;,\;V\;\right)$. Last, $\hat{BF}$ can be derived as below:
  $$\begin{array}{l} \hat{Var^{**}}\left(\tilde{X}|{\;\text{W }}_{s},{\;\text{V }}_{s}\right)=\frac{1}{2}\left(\hat{Var_{1}^{**}}\left(\tilde{X}|{\;\text{W }}_{s},{\;\text{V }}_{s}\right)+\hat{Var_{2}^{**}}\left(\tilde{X}|{\;\text{W }}_{s},{\;\text{V }}_{s}\right)\right) \end{array}$$
  $$\begin{array}{l} \hat{BF}=1-\frac{\hat{Var^{**}}\left(\tilde{X}|{\;\text{W }}_{s},{\;\text{V }}_{s}\right)-{\tilde{\sigma }}_{x}^{2}}{\hat{Var}\left(\tilde{X}|\;\text{V }\right)-{\tilde{\sigma }}_{x}^{2}}. \end{array}$$

With $\hat{BF}$, we have ${\hat{X}}_{2i}={\hat{X}}_{1i}/\hat{BF}$. We can run a regression of ${\hat{X}}_{2}$ on self-reported food frequency questionnaire data (*Q*) and ***V*** to build calibration equation using the *n*_2_ calibration samples to estimate the parameter


$$\begin{array}{l} {\hat{\gamma }}_{2}={\left\{\overset{n_{1}+n_{2}}{\underset{i=n_{1}+1}{\sum }}{\left(1,Q_{i},{\;\text{V }}_{i}^{⊤}\right)}^{⊤}\left(1,Q_{i},{\;\text{V }}_{i}^{⊤}\right)\right\}}^{-1}\left\{\overset{n_{1}+n_{2}}{\underset{i=n_{1}+1}{\sum }}{\left(1,Q_{i},{\;\text{V }}_{i}^{⊤}\right)}^{⊤}{\hat{X}}_{2i}\right\}. \end{array}$$

Using the Stage 3 sample, we then estimate *Z* as ${\hat{Z}}_{2i}=\left(1,Q_{i},{\;V\;}_{i}^{⊤}\right){\hat{\gamma }}_{2}$ for *i* = *n*_1_ + *n*_2_ + 1, ⋯ , *n*_1_ + *n*_2_ + *n*_3_. Finally, we estimate the association of *Z* with the time-to-event endpoint (T^*^, Δ) by solving the score equation for Cox model:


$$\begin{array}{l} 0=\sum_{i=n_{1}+n_{2}+1}^{n_{1}+n_{2}+n_{3}}\int_{0}^{\tau }[\left(\begin{array}{c} {\hat{Z}}_{2i}\\ {\text{V}}_{i} \end{array}\right)\\ \;-\sum_{j}\frac{Y_{j}(t)\exp\left\{({\hat{Z}}_{2j},{\text{V}}_{j}^{⊤})\theta \right\}}{\sum_{k}Y_{k}(t)\exp\left\{({\hat{Z}}_{2k},{\text{V}}_{k}^{⊤})\theta \right\}}\left(\begin{array}{c} {\hat{Z}}_{2j}\\ {\text{V}}_{j} \end{array}\right)]dN_{i}(t). \end{array}$$

For method 2 to work, we can relax Equation (3) to that the conditional mean 𝔼[*Z*|***W***,***V***] from sample 2 is the same as the conditional mean 𝔼[*X*|***W***,***V***] from sample 1.

### 3.3. Method 3: three-step with self-reported data

If the self-reported data *Q* from the feeding study is accessible and we presume that the distributions of (*Q*|*Z*,***V***) remain consistent between the controlled feeding study and the cohort, the bias in the naive estimator can be rectified by simply incorporating *Q* into the biomarker development equation. This is because the inclusion of *Q* ensures that 𝔼[Ẑ|*Q*,***V***] = 𝔼[𝔼[*Z*|*Q*,***V***,***W***]|*Q*,***V***] = 𝔼[*Z*|*Q*,***V***].

The sequence of the first method remains unchanged, but in the first step of the regression model, the log-transformed self-reported food frequency questionnaire data (*Q*) is included. Specifically, for the first step, the predictors ***W***, ***V***, and *Q* are utilized to construct the biomarker. Following this, in the second step, we employ ***W***, ***V***, and *Q* to estimate *Z*. Lasso, SCAD, and RF, as previously described, are all applied in Method 3 for variable selection and effect estimation in high-dimensional statistical inference, considering both direct selection and post-selection.

With the estimated ${\hat{\beta }}_{3}$ from the first step, ${\hat{X}}_{3i}=\left(1,{\;W\;}_{i},Q_{i},{\;V\;}_{i}^{⊤}\right){\hat{\beta }}_{3}$, we can execute a regression of ${\hat{X}}_{3i}$ on the self-reported food frequency questionnaire data (*Q*) and ***V*** to construct a calibration equation using the *n*_2_ calibration samples to estimate the parameter


$$\begin{array}{l} {\hat{\gamma }}_{3}={\left\{\overset{n_{1}+n_{2}}{\underset{i=n_{1}+1}{\sum }}{\left(1,Q_{i},{\;\text{V }}_{i}^{⊤}\right)}^{⊤}\left(1,Q_{i},{\;\text{V }}_{i}^{⊤}\right)\right\}}^{-1}\left\{\overset{n_{1}+n_{2}}{\underset{i=n_{1}+1}{\sum }}{\left(1,Q_{i},{\;\text{V }}_{i}^{⊤}\right)}^{⊤}{\hat{X}}_{3i}\right\}. \end{array}$$

Using the Stage 3 sample, we then estimate *Z* as ${\hat{Z}}_{3i}=\left(1,Q_{i},{\;V\;}_{i}^{⊤}\right){\hat{\gamma }}_{3}$ for *i* = *n*_1_ + *n*_2_ + 1, ⋯ , *n*_1_ + *n*_2_ + *n*_3_. Finally, we estimate the association of *Z* with the time-to-event endpoint (T^*^, Δ) by solving the score equation for Cox model:


$$\begin{array}{l} 0=\sum_{i=n_{1}+n_{2}+1}^{n_{1}+n_{2}+n_{3}}\int_{0}^{\tau }[\left(\begin{array}{c} {\hat{Z}}_{3i}\\ {\text{V}}_{i} \end{array}\right)\\ \;-\sum_{j}\frac{Y_{j}(t)\exp\left\{({\hat{Z}}_{3j},{\text{V}}_{j}^{⊤})\theta \right\}}{\sum_{k}Y_{k}(t)\exp\left\{({\hat{Z}}_{3k},{\text{V}}_{k}^{⊤})\theta \right\}}\left(\begin{array}{c} {\hat{Z}}_{3j}\\ {\text{V}}_{j} \end{array}\right)]dN_{i}(t). \end{array}$$

For method 3 to work, we can relax Equation (3) to that the conditional mean 𝔼[*Z*|***W***, *Q*,***V***] from sample 2 is the same as the conditional mean 𝔼[*X*|***W***, *Q*,***V***] from sample 1.

### 3.4. Method 4: direct estimation

We build the estimating equation by regressing $\tilde{X}$ on *Q* and ***V*** in the first step and directly apply it to the third step. Then we build the calibration equation using the feeding study by regressing $\tilde{X}$ on ***V*** and *Q* and use the calibration equation to predict *Z* and perform a Cox regression of *Y* on *Z* and ***V*** in the full cohort to estimate the association parameter. In other words, we have


$$\begin{array}{l} {\hat{\gamma }}_{4}={\left\{\overset{n_{1}+n_{2}}{\underset{i=n_{1}+1}{\sum }}{\left(1,Q_{i},{\;\text{V }}_{i}^{⊤}\right)}^{⊤}\left(1,Q_{i},{\;\text{V }}_{i}^{⊤}\right)\right\}}^{-1}\left\{\overset{n_{1}+n_{2}}{\underset{i=n_{1}+1}{\sum }}{\left(1,Q_{i},{\;\text{V }}_{i}^{⊤}\right)}^{⊤}{\tilde{X}}_{i}\right\}, \end{array}$$

${Ẑ}_{4i}=\left(1,Q_{i},{\;V\;}_{i}\right){\hat{\gamma }}_{4}$ and ${\hat{\theta }}_{4}$ by solving estimating equations


$$\begin{array}{l} 0=\sum_{i=n_{1}+n_{2}+1}^{n_{1}+n_{2}+n_{3}}\int_{0}^{\tau }[\left(\begin{array}{c} {\hat{Z}}_{4i}\\ {\text{V}}_{i} \end{array}\right)\\ \;-\sum_{j}\frac{Y_{j}(t)\exp\left\{({\hat{Z}}_{4j},{\text{V}}_{j}^{⊤})\theta \right\}}{\sum_{k}Y_{k}(t)\exp\left\{({\hat{Z}}_{4k},{\text{V}}_{k}^{⊤})\theta \right\}}\left(\begin{array}{c} {\hat{Z}}_{4j}\\ {\text{V}}_{j} \end{array}\right)]dN_{i}(t). \end{array}$$

For method 4 to work, we can relax Equation (3) to that the conditional mean 𝔼[*Z*|*Q*,***V***] from sample 3 is the same as the conditional mean 𝔼[*X*|*Q*,***V***] from sample 1.

## 4. Simulation

We simulate data with varying levels of sparsity, effect size, and shape within the context of high-dimensional statistical inference. Our goal is to investigate how the sparsity, effect size, and shape among different measurements influence the bias and variance of various estimators. We compare the bias, empirical standard deviation (SD), estimated standard error (SE), and coverage rate for a nominal 95% confidence interval (CR) across different sample sizes, effect shapes, effect sizes, and correlation structures. Here the CR is computed from the asymptotic SE formula as shown in the Theorem 1 of (20) with the term ${\hat{\Sigma }}_{\gamma k}$ estimated from 100 Bootstrap samples using data from the first two samples given that there is no closed-form variance formula for Σ_γ*k*_ when *W* is of high-dimension. We examine both scenarios, with and without penalties applied to the personal characteristics *V* during the first stage of penalized regression. Time-to-event outcomes are generated using the Cox model.


$$\begin{array}{l} \left(Z,V\right)\sim N\left(0,\left(\begin{array}{cc} 1-\sigma_{x}^{2} & \rho \\ \rho & 1 \end{array}\right)\right),\\ \;\text{W }=b_{0}+b_{1}X+b_{2}V+\epsilon_{w},\\ X=Z+\epsilon_{x},\\ \tilde{X}=X+\epsilon_{\tilde{X}},\\ Q=a_{0}+a_{1}Z+a_{2}V+\epsilon_{q},\\ \lambda \left(t|Z,X,V,\;\text{W },Q\right)=\lambda \left(t|Z,V\right)=\lambda_{0}\left(t\right)\exp\left(\theta_{z}Z+\theta_{v}V\right), \end{array}$$

where *Z*, *V*, *X*, and *Q* ∈ ℝ, while ***W*** ∈ ℝ^*p*^ is high-dimensional. In this study, ϵ_*x*_ and ϵ_*q*_ are independently sampled from normal distributions with mean zero and standard deviations σ_*x*_ and σ_*q*_. The censoring time is sampled from a mixture of a uniform distribution Unif(0, 10) and a point mass at 10, with equal probability. Three settings are considered: (i) baseline setting, (ii) weak biomarker effect and strong self-reported data effect, and (iii) strong biomarker effect. We experiment with three sparsity levels of ***W*** (2, 5, and 10), and consider two different patterns of the effect size for ***W***: equivalent and random. More details on the parameter settings can be found in Supplementary material (Section 1.1).

The bias, mean estimated standard error (SE), empirical standard deviation (SD), and coverage rate (CR) of 95% nominal confidence interval for all four methods from 100 simulations are listed in Tables 1, 2 with Lasso penalized regression. The performance of our proposed Method 2 varies under different scenarios. Post selection and direct selection are both performed for variable selection. The results are for the ones forcing personal characteristics (*V*) in the model while the results for not forcing *V* in the model can be found in Supplementary Tables 2, 3.

**Table 1:** Simulation results with direct Lasso selection forcing personal characteristics in the model.

**Pattern**	**Sparsity**		**Setting 1**				**Setting 2**				**Setting 3**			
		**Method**	**Bias**	**SD**	**SE**	**CR**	**Bias**	**SD**	**SE**	**CR**	**Bias**	**SD**	**SE**	**CR**
Same	2	1	0.69	0.844	0.867	0.94	0.53	0.469	0.461	0.85	0.28	0.453	0.462	0.94
		2	0.03	0.298	0.362	0.96	0.02	0.187	0.208	0.97	0.02	0.276	0.288	0.96
		3	−0.02	0.249	0.287	0.96	−0.02	0.155	0.182	0.97	−0.02	0.245	0.276	0.97
		4	−0.01	0.280	0.355	0.94	−0.02	0.158	0.194	0.95	−0.01	0.280	0.355	0.94
	5	1	0.30	5.454	1.294	0.95	0.66	0.761	0.596	0.87	0.34	0.613	0.528	0.95
		2	−0.06	0.375	0.402	0.95	−0.01	0.209	0.215	0.96	0.00	0.287	0.297	0.95
		3	−0.03	0.259	0.300	0.97	−0.02	0.167	0.186	0.98	−0.03	0.264	0.283	0.96
		4	−0.01	0.280	0.355	0.94	−0.02	0.158	0.194	0.95	−0.01	0.280	0.355	0.94
	10	1	78.75	776.634	107.201	0.94	0.89	1.328	0.819	0.88	0.38	0.662	0.625	0.94
		2	−3.72	36.126	2.578	0.95	−0.07	0.175	0.229	0.99	−0.04	0.236	0.307	0.95
		3	−0.03	0.259	0.312	0.95	−0.03	0.160	0.192	0.97	−0.03	0.257	0.292	0.94
		4	−0.01	0.280	0.355	0.94	−0.02	0.158	0.194	0.95	−0.01	0.280	0.355	0.94
Random	2	1	0.63	0.770	0.760	0.90	0.52	0.420	0.459	0.88	0.23	0.399	0.440	0.93
		2	0.04	0.369	0.324	0.96	0.04	0.212	0.217	0.97	0.02	0.268	0.289	0.94
		3	0.00	0.356	0.291	0.96	−0.01	0.178	0.182	0.97	0.04	0.746	0.278	0.96
		4	−0.01	0.258	0.324	0.97	0.00	0.177	0.191	0.96	−0.01	0.258	0.324	0.97
	5	1	0.69	0.708	0.850	0.88	0.57	0.459	0.506	0.9	0.27	0.381	0.474	0.97
		2	0.03	0.258	0.350	1.00	0.04	0.184	0.225	0.97	0.03	0.242	0.304	0.99
		3	−0.01	0.227	0.303	0.97	0.00	0.149	0.194	0.96	−0.01	0.219	0.290	0.97
		4	0.01	0.250	0.323	0.97	0.01	0.161	0.206	0.98	0.01	0.250	0.323	0.97
	10	1	0.92	1.022	0.955	0.87	0.61	0.503	0.513	0.78	0.37	0.520	0.486	0.89
		2	0.07	0.322	0.349	0.93	0.03	0.206	0.214	0.94	0.07	0.304	0.294	0.91
		3	0.04	0.287	0.296	0.95	−0.01	0.172	0.183	0.94	0.03	0.278	0.281	0.95
		4	0.05	0.309	0.330	0.95	−0.01	0.173	0.188	0.93	0.05	0.309	0.330	0.95

**Table 2:** Simulation results with post Lasso selection forcing personal characteristics in the model.

**Pattern**	**Sparsity**		**Setting 1**				**Setting 2**				**Setting 3**			
		**Method**	**Bias**	**SD**	**SE**	**CR**	**Bias**	**SD**	**SE**	**CR**	**Bias**	**SD**	**SE**	**CR**
Same	2	1	0.46	0.813	0.658	0.93	0.36	0.421	0.375	0.91	0.16	0.376	0.387	0.94
		2.1	−0.20	0.226	0.217	0.79	−0.15	0.154	0.160	0.76	−0.103	0.208	0.216	0.90
		2.2	0.18	0.480	0.436	0.93	0.10	0.262	0.248	0.93	0.07	0.308	0.322	0.95
		2.3	0.02	0.374	0.329	0.94	0.00	0.219	0.203	0.94	−0.01	0.266	0.273	0.94
		3	−0.01	0.260	0.404	0.97	−0.02	0.167	0.190	0.97	0.00	0.274	0.336	0.97
		4	−0.01	0.280	0.355	0.94	−0.02	0.158	0.194	0.95	−0.01	0.280	0.355	0.94
	5	1	0.39	0.858	1.286	0.95	0.41	0.592	0.434	0.92	0.18	0.448	0.417	0.95
		2.1	−0.24	0.181	0.296	0.80	−0.18	0.131	0.169	0.81	−0.115	0.206	0.217	0.89
		2.2	0.09	0.470	0.792	0.96	0.11	0.319	0.267	0.91	0.06	0.333	0.335	0.94
		2.3	−0.09	0.277	0.524	0.95	−0.04	0.222	0.205	0.97	−0.04	0.259	0.269	0.93
		3	−0.02	0.280	0.348	0.96	−0.02	0.187	0.190	0.98	−0.01	0.303	0.303	0.97
		4	−0.01	0.280	0.355	0.94	−0.02	0.158	0.194	0.95	−0.01	0.280	0.355	0.94
	10	1	0.49	1.302	1.341	0.95	0.62	1.157	0.563	0.89	0.26	0.542	0.501	0.93
		2.1	−0.32	0.406	0.316	0.73	−0.27	0.199	0.191	0.72	−0.170	0.174	0.222	0.89
		2.2	0.12	0.609	0.771	0.95	0.16	0.528	0.317	0.93	0.08	0.355	0.374	0.93
		2.3	−0.14	0.285	0.407	0.94	−0.08	0.321	0.216	0.93	−0.06	0.269	0.275	0.92
		3	−0.03	0.269	0.334	0.95	−0.03	0.181	0.196	0.98	−0.03	0.256	0.304	0.96
		4	−0.01	0.280	0.355	0.94	−0.02	0.158	0.194	0.95	−0.01	0.280	0.355	0.94
Random	2	1	0.46	0.740	0.653	0.93	0.36	0.373	0.381	0.87	0.16	0.384	0.379	0.94
		2.1	−0.18	0.263	0.221	0.75	−0.15	0.155	0.162	0.79	−0.102	0.214	0.211	0.92
		2.2	0.19	0.554	0.438	0.94	0.11	0.255	0.257	0.94	0.08	0.336	0.321	0.95
		2.3	0.04	0.432	0.325	0.95	0.00	0.208	0.209	0.95	0.00	0.293	0.272	0.94
		3	−0.05	0.390	0.335	0.97	0.00	0.210	0.190	0.96	−0.04	0.311	0.301	0.96
		4	−0.01	0.258	0.324	0.97	0.00	0.177	0.191	0.96	−0.01	0.258	0.324	0.97
	5	1	0.42	0.532	0.702	0.94	0.37	0.353	0.413	0.90	0.16	0.323	0.410	0.97
		2.1	−0.20	0.184	0.251	0.85	−0.15	0.142	0.173	0.81	−0.102	0.184	0.232	0.93
		2.2	0.14	0.335	0.479	0.98	0.11	0.223	0.276	0.97	0.07	0.266	0.345	0.98
		2.3	0.00	0.268	0.349	0.95	0.00	0.184	0.221	0.97	0.00	0.232	0.290	0.98
		3	−0.01	0.241	0.480	0.96	0.00	0.152	0.201	0.96	−0.01	0.232	0.317	0.95
		4	0.01	0.250	0.323	0.97	0.01	0.161	0.206	0.98	0.01	0.250	0.323	0.97
	10	1	0.57	0.792	0.675	0.89	0.37	0.383	0.388	0.86	0.23	0.414	0.401	0.91
		2.1	−0.20	0.269	0.244	0.77	−0.16	0.158	0.161	0.75	−0.077	0.221	0.220	0.88
		2.2	0.23	0.492	0.442	0.94	0.10	0.249	0.253	0.92	0.12	0.336	0.329	0.91
		2.3	0.01	0.297	0.319	0.96	−0.02	0.188	0.201	0.94	0.03	0.277	0.277	0.94
		3	0.04	0.285	0.325	0.95	0.00	0.181	0.190	0.96	0.04	0.282	0.300	0.94
		4	0.05	0.309	0.330	0.95	−0.01	0.173	0.188	0.93	0.05	0.309	0.330	0.95

In general, the post selection methods perform slightly better than the direct-selection methods with lower SDs and SEs. For a few settings, the direct selection approach does not perform stably in terms of bias and SD. The direct selection not forcing the inclusion of personal characteristics showed more stable results compared with direct selection forcing the inclusion of personal characteristics for Method 2 but the variance is larger in general for all other methods. For the post selection approach, the performances of the three variance estimation methods, are shown as 2.1 (*K*-fold cross-validation), 2.2 (modified variance estimator), and 2.3 (RCV) in Tables 2, **4**, **6**. Method 2.3 (RCV estimation under post selection within Method 2) performs the best among all three approaches across different settings and patterns. Some key advantages of Method 2.3 include lower bias and smaller standard deviations (SD) and standard errors (SE), along with good coverage rates (CR).

Tables 1, 2 shows the results using Lasso penalized regression when forcing personal characteristics in the model. As the sparsity level increases, the performance of most methods seems to degrade, with higher biases and lower coverage rates. Methods 3 and 4 demonstrate good performances in most of the settings. However, when the strength of the biomarker is strong and the strength of FFQ is relatively weak (i,e., Setting 3), we can see Method 2 generally generated the most efficient result compared with Methods 3 and 4. When we have strong biomarker effects (Setting 3), Method 2.3 outperforms the other methods.

Tables 3, 4 present the results for SCAD penalized regression when personal characteristics are forced into the model. Corresponding results without forcing personal characteristics can be found in Supplementary Tables 4, 5. When comparing SCAD with Lasso, we can observe a similar trend in terms of bias control and standard deviation (SD) across various effect size patterns and settings. In addition, when comparing the three approaches for variance estimation in constructing the bias factor (BF) using Method 2 with post selection, the RCV approach continues to outperform the others in controlling bias and providing the most efficient results. With direct selection using SCAD in Method 2, the bias is generally well-controlled, and the confidence rate (CR) is promising when personal characteristics are not fixed for variable filtering. These results are comparable to those obtained with Lasso. However, when variables are post selected, SCAD's performance is not as strong as Lasso's. This is particularly noticeable in scenarios with large sparsity, where SCAD struggles to control bias effectively. In summary, Lasso demonstrates superior performance in variance estimation and bias control when compared to SCAD.

**Table 3:** Simulation results for direct SCAD selection forcing personal characteristics in the model.

**Pattern**	**Sparsity**		**Setting 1**				**Setting 2**				**Setting 3**			
		**Method**	**Bias**	**SD**	**SE**	**CR**	**Bias**	**SD**	**SE**	**CR**	**Bias**	**SD**	**SE**	**CR**
Same	2	1	0.53	1.219	0.715	0.94	0.39	0.463	0.384	0.89	0.17	0.359	0.398	0.94
		2	−0.02	0.356	0.277	0.95	−0.04	0.179	0.180	0.95	−0.04	0.230	0.252	0.94
		3	−0.02	0.248	0.306	0.97	−0.01	0.176	0.185	0.96	−0.02	0.266	0.290	0.96
		4	−0.01	0.280	0.355	0.94	−0.02	0.158	0.194	0.95	−0.01	0.280	0.355	0.94
	5	1	1.00	2.047	2.359	0.91	1.48	8.942	0.734	0.91	0.37	1.263	0.538	0.95
		2	−0.12	0.255	0.387	0.93	−0.07	0.257	0.201	0.93	−0.06	0.326	0.262	0.91
		3	−0.02	0.259	0.317	0.97	−0.03	0.156	0.193	0.98	−0.01	0.331	0.319	0.96
		4	−0.01	0.280	0.355	0.94	−0.02	0.158	0.194	0.95	−0.01	0.280	0.355	0.94
	10	1	3.15	5.880	8.839	0.90	1.56	2.116	2.040	0.82	0.55	0.927	0.971	0.92
		2	−8.89	62.117	1.686	0.88	−0.12	0.239	0.357	0.88	−0.12	0.230	0.297	0.93
		3	−0.02	0.272	0.338	0.95	−0.02	0.165	0.197	0.95	−0.02	0.272	0.312	0.95
		4	−0.01	0.280	0.355	0.94	−0.02	0.158	0.194	0.95	−0.01	0.280	0.355	0.94
Random	2	1	0.42	0.572	0.623	0.93	0.39	0.371	0.380	0.86	0.16	0.357	0.383	0.93
		2	−0.03	0.281	0.281	0.95	−0.02	0.179	0.190	0.96	−0.03	0.242	0.257	0.94
		3	−0.25	2.306	0.299	0.97	0.00	0.190	0.185	0.95	−0.18	1.627	0.284	0.96
		4	−0.01	0.258	0.324	0.97	0.00	0.177	0.191	0.96	−0.01	0.258	0.324	0.97
	5	1	0.52	0.618	0.742	0.89	0.43	0.356	0.424	0.91	0.20	0.339	0.421	0.97
		2	−0.03	0.231	0.300	0.97	−0.02	0.162	0.196	0.98	−0.02	0.221	0.265	0.98
		3	−0.01	0.226	0.311	0.97	0.00	0.149	0.197	0.96	−0.01	0.221	0.299	0.96
		4	0.01	0.250	0.323	0.97	0.01	0.161	0.206	0.98	0.01	0.250	0.323	0.97
	10	1	0.81	1.084	0.923	0.87	0.51	0.499	0.471	0.81	0.29	0.459	0.446	0.89
		2	−0.01	0.291	0.310	0.94	−0.06	0.162	0.185	0.95	0.01	0.265	0.259	0.92
		3	0.04	0.328	0.309	0.95	−0.01	0.171	0.185	0.93	0.03	0.292	0.294	0.96
		4	0.05	0.309	0.330	0.95	−0.01	0.173	0.188	0.93	0.05	0.309	0.330	0.95

**Table 4:** Simulation results for post SCAD selection forcing personal characteristics in the model.

**Pattern**	**Sparsity**		**Setting 1**				**Setting 2**				**Setting 3**			
		**Method**	**Bias**	**SD**	**SE**	**CR**	**Bias**	**SD**	**SE**	**CR**	**Bias**	**SD**	**SE**	**CR**
Same	2	1	0.49	0.940	1.289	0.92	0.46	0.591	0.514	0.86	0.18	0.420	0.443	0.95
		2.1	−0.23	0.180	0.227	0.78	−0.18	0.154	0.166	0.74	−0.12	0.201	0.208	0.87
		2.2	0.15	0.393	0.470	0.94	0.11	0.293	0.251	0.93	0.07	0.308	0.321	0.95
		2.3	0.08	0.500	2.590	0.93	0.01	0.268	0.670	0.93	0.02	0.329	1.304	0.94
		3	−0.01	0.259	0.331	0.97	−0.01	0.171	0.194	0.96	0.00	0.262	0.315	0.97
		4	−0.01	0.280	0.355	0.94	−0.02	0.158	0.194	0.95	−0.01	0.280	0.355	0.94
	5	1	0.46	2.725	11.428	0.93	0.42	2.520	3.700	0.93	0.25	0.732	0.667	0.91
		2.1	−0.29	0.312	0.309	0.71	−0.18	0.175	0.201	0.79	−0.13	0.237	0.224	0.86
		2.2	0.46	3.503	0.759	0.95	0.17	0.884	0.406	0.93	0.10	0.464	0.361	0.96
		2.3	0.41	4.576	2.323	0.91	−0.21	1.818	1.132	0.87	−0.01	0.315	0.650	0.94
		3	−0.01	0.322	0.352	0.96	−0.01	0.229	0.195	0.98	0.01	0.450	0.462	0.97
		4	−0.01	0.280	0.355	0.94	−0.02	0.158	0.194	0.95	−0.01	0.280	0.355	0.94
	10	1	1.12	2.994	8.838	0.94	1.08	3.364	3.596	0.88	0.44	0.690	1.498	0.90
		2.1	−0.43	0.773	0.858	0.83	−0.27	0.319	0.307	0.78	−0.19	0.208	0.268	0.86
		2.2	0.16	0.628	1.148	0.96	0.18	0.534	0.357	0.95	0.09	0.334	0.436	0.95
		2.3	0.05	0.629	2.045	0.95	−0.01	0.893	2.983	0.93	0.10	1.633	1.124	0.95
		3	−0.02	0.276	0.412	0.94	−0.02	0.174	0.200	0.95	−0.02	0.269	0.357	0.95
		4	−0.01	0.280	0.355	0.94	−0.02	0.158	0.194	0.95	−0.01	0.280	0.355	0.94
Random	2	1	0.45	0.641	2.098	0.97	0.44	0.717	0.674	0.87	0.20	0.614	0.432	0.96
		2.1	−0.25	0.191	0.254	0.72	−0.18	0.147	0.161	0.77	−0.13	0.198	0.227	0.86
		2.2	0.06	0.742	0.431	0.96	0.11	0.263	0.258	0.91	0.07	0.341	0.323	0.91
		2.3	0.06	0.429	0.839	0.94	0.03	0.243	0.321	0.95	0.01	0.286	0.362	0.94
		3	−0.03	0.288	0.348	0.96	0.02	0.294	0.191	0.96	−0.03	0.313	0.309	0.95
		4	−0.01	0.258	0.324	0.97	0.00	0.177	0.191	0.96	−0.01	0.258	0.324	0.97
	5	1	0.64	1.151	3.383	0.92	0.46	0.497	0.657	0.90	0.21	0.381	0.476	0.97
		2.1	−0.24	0.183	0.433	0.77	−0.18	0.150	0.181	0.75	−0.12	0.181	0.222	0.91
		2.2	0.15	0.342	0.504	0.97	0.11	0.214	0.278	0.97	0.07	0.268	0.351	0.99
		2.3	0.06	0.409	0.811	0.97	0.02	0.231	0.327	0.98	0.02	0.229	0.364	0.99
		3	−0.01	0.230	0.476	0.97	0.00	0.162	0.205	0.96	−0.01	0.231	0.324	0.95
		4	0.01	0.250	0.323	0.97	0.01	0.161	0.206	0.98	0.01	0.250	0.323	0.97
	10	1	0.96	1.940	4.993	0.92	0.84	2.196	1.202	0.86	0.35	0.762	0.525	0.95
		2.1	−0.24	0.247	0.300	0.73	−0.19	0.171	0.175	0.78	−0.10	0.216	0.217	0.84
		2.2	0.25	0.480	0.465	0.88	0.11	0.256	0.262	0.94	0.12	0.339	0.337	0.93
		2.3	0.24	1.034	1.989	0.93	0.04	0.348	0.374	0.92	0.08	0.442	0.338	0.92
		3	0.05	0.313	0.346	0.94	0.00	0.192	0.192	0.94	0.04	0.289	0.318	0.95
		4	0.05	0.309	0.330	0.95	−0.01	0.173	0.188	0.93	0.05	0.309	0.330	0.95

RF offers an alternative approach for constructing a biomarker prediction model in the second stage. Tables 5, 6 display the results obtained using RF. When the 10 most important variables are directly selected with RF, the estimated bias is considerably large in most scenarios. However, when post selection is applied to variables using RF, results with variance estimation approaches 2.2 and 2.3 both exhibit small bias and promising confidence rates (CR). These outcomes are comparable to those achieved with Lasso for Method 2 using the RCV estimation (2.3). In summary, while RF does not provide accurate estimations of associated parameters when using direct selection, its performance is similar to Lasso when post selection is employed.

**Table 5:** Simulation results for direct RF selection not forcing personal characteristics in the model.

**Pattern**	**Sparsity**		**Setting 1**				**Setting 2**				**Setting 3**			
		**Method**	**Bias**	**SD**	**SE**	**CR**	**Bias**	**SD**	**SE**	**CR**	**Bias**	**SD**	**SE**	**CR**
Same	2	1	1.08	20.056	42.699	0.91	2.29	1.361	2.066	0.90	6.28	17.557	22.674	0.91
		2	−0.07	7.706	4.795	0.93	0.35	0.528	0.645	0.93	3.56	17.387	15.925	0.91
		3	1.62	1.506	2.256	0.91	1.03	0.724	0.785	0.84	1.94	2.153	3.978	0.9
		4	−0.01	0.280	0.355	0.94	−0.02	0.158	0.194	0.95	−0.01	0.280	0.355	0.94
	5	1	2.77	3.167	5.799	0.90	1.90	1.236	1.366	0.76	1.67	1.840	2.236	0.88
		2	0.17	0.753	1.028	0.93	0.00	0.242	0.324	0.98	0.62	1.384	0.919	0.87
		3	1.16	1.282	1.335	0.89	0.69	0.520	0.587	0.84	0.87	0.876	1.067	0.93
		4	−0.01	0.280	0.355	0.94	−0.02	0.158	0.194	0.95	−0.01	0.280	0.355	0.94
	10	1	2.34	2.588	4.669	0.88	2.40	1.475	2.011	0.86	1.18	1.374	1.195	0.88
		2	−0.18	3.913	0.618	0.95	−0.13	0.254	0.393	0.93	0.07	0.370	0.463	0.95
		3	0.99	1.141	1.293	0.87	0.60	0.486	0.641	0.90	0.67	0.765	0.835	0.89
		4	−0.01	0.280	0.355	0.94	−0.02	0.158	0.194	0.95	−0.01	0.280	0.355	0.94
Random	2	1	−2.68	31.746	61.244	0.92	1.29	25.138	109.942	0.86	−80.83	641.046	101.489	0.94
		2	−1.85	9.158	14.467	0.95	−0.89	5.915	19.942	0.88	19.35	184.338	46.897	0.9
		3	3.53	6.489	25.208	0.98	2.56	23.515	24.412	0.92	−0.63	42.632	40.913	0.94
		4	−0.01	0.258	0.324	0.97	0.00	0.177	0.191	0.96	−0.01	0.258	0.324	0.97
	5	1	32.47	339.650	88.785	0.91	−0.50	66.632	120.634	0.88	−16.21	142.496	168.673	0.92
		2	8.23	83.762	26.403	0.92	−0.06	8.969	53.692	0.91	−0.43	22.138	67.965	0.94
		3	−1.26	20.206	24.392	0.91	1.39	23.664	33.013	0.82	1.70	39.330	52.827	0.87
		4	0.01	0.250	0.323	0.97	0.01	0.161	0.206	0.98	0.01	0.250	0.323	0.97
	10	1	4.92	81.304	82.072	0.89	6.89	58.844	71.207	0.89	−5.83	125.518	375.281	0.88
		2	0.01	7.096	21.133	0.98	48.46	496.413	595.578	0.90	2.63	31.842	43.618	0.91
		3	−14.44	127.206	13.957	0.89	2.56	4.880	172.054	0.84	4.08	17.941	31.275	0.89
		4	0.05	0.309	0.330	0.95	−0.01	0.173	0.188	0.93	0.05	0.309	0.330	0.95

**Table 6:** Simulation results for post RF selection not forcing personal characteristics in the model.

**Pattern**	**Sparsity**		**Setting 1**				**Setting 2**				**Setting 3**			
		**Method**	**Bias**	**SD**	**SE**	**CR**	**Bias**	**SD**	**SE**	**CR**	**Bias**	**SD**	**SE**	**CR**
Same	2	1	0.47	0.606	0.655	0.88	0.41	0.383	0.394	0.83	0.17	0.373	0.391	0.93
		2.1	−0.09	0.217	0.248	0.90	−0.09	0.153	0.167	0.91	−0.07	0.218	0.238	0.94
		2.2	0.00	0.275	0.306	0.97	0.04	0.199	0.217	0.96	−0.02	0.246	0.264	0.95
		2.3	0.00	0.279	0.324	0.97	−0.01	0.189	0.205	0.96	0.00	0.259	0.275	0.95
		3	−0.02	0.260	0.308	0.96	−0.01	0.170	0.193	0.97	−0.01	0.262	0.291	0.95
		4	−0.01	0.280	0.355	0.94	−0.02	0.158	0.194	0.95	−0.01	0.280	0.355	0.94
	5	1	0.72	1.298	0.974	0.90	0.61	0.569	0.564	0.85	0.25	0.483	0.463	0.92
		2.1	−0.10	0.266	0.297	0.91	−0.14	0.161	0.187	0.84	−0.06	0.236	0.253	0.94
		2.2	0.02	0.354	0.384	0.97	0.06	0.229	0.263	0.94	−0.01	0.262	0.282	0.94
		2.3	0.17	1.820	0.366	0.96	−0.07	0.180	0.209	0.96	0.01	0.285	0.299	0.96
		3	0.00	0.293	0.345	0.98	0.00	0.183	0.201	0.97	0.03	0.365	0.337	0.93
		4	−0.01	0.280	0.355	0.94	−0.02	0.158	0.194	0.95	−0.01	0.280	0.355	0.94
	10	1	1.02	1.184	2.059	0.91	1.20	1.336	1.205	0.86	0.59	0.736	0.818	0.90
		2.1	−0.17	0.229	0.412	0.92	−0.24	0.299	0.244	0.73	−0.05	0.269	0.317	0.96
		2.2	0.04	0.338	0.646	0.97	0.17	0.416	0.444	0.92	0.04	0.309	0.379	0.95
		2.3	−0.01	0.335	0.517	0.95	−0.07	0.268	0.275	0.90	0.09	0.372	0.431	0.91
		3	0.06	0.524	0.395	0.94	0.01	0.188	0.213	0.97	0.25	1.677	13.383	0.93
		4	−0.01	0.280	0.355	0.94	−0.02	0.158	0.194	0.95	−0.01	0.280	0.355	0.94
Random	2	1	0.43	0.570	0.651	0.91	0.39	0.367	0.395	0.88	0.15	0.341	0.381	0.93
		2.1	−0.09	0.230	0.248	0.95	−0.07	0.164	0.177	0.92	−0.06	0.222	0.238	0.97
		2.2	0.03	0.313	0.328	0.96	0.04	0.203	0.224	0.97	0.00	0.254	0.275	0.95
		2.3	−0.02	0.282	0.308	0.97	0.01	0.199	0.210	0.96	−0.01	0.251	0.269	0.97
		3	0.05	0.740	0.316	0.97	0.01	0.188	0.194	0.96	−0.25	2.308	0.293	0.96
		4	−0.01	0.258	0.324	0.97	0.00	0.177	0.191	0.96	−0.01	0.258	0.324	0.97
	5	1	0.68	1.701	0.743	0.93	0.41	0.350	0.421	0.87	0.18	0.325	0.408	0.98
		2.1	−0.08	0.380	0.257	0.91	−0.07	0.146	0.177	0.96	−0.05	0.202	0.249	0.98
		2.2	0.08	0.459	0.351	0.97	0.05	0.189	0.228	0.96	0.01	0.232	0.290	1.00
		2.3	−0.04	0.238	0.461	0.95	0.00	0.176	0.210	0.99	−0.01	0.223	0.280	1.00
		3	0.01	0.236	0.334	0.97	0.02	0.159	0.204	0.97	0.00	0.225	0.305	0.96
		4	0.01	0.250	0.323	0.97	0.01	0.161	0.206	0.98	0.01	0.250	0.323	0.97
	10	1	0.70	2.558	1.365	0.87	0.45	0.443	0.425	0.84	0.26	0.431	0.421	0.92
		2.1	−0.05	0.377	0.310	0.87	−0.11	0.167	0.174	0.85	−0.02	0.247	0.240	0.91
		2.2	0.13	0.507	0.444	0.94	0.04	0.213	0.221	0.94	0.05	0.291	0.284	0.91
		2.3	0.06	0.797	0.579	0.87	−0.03	0.206	0.195	0.93	0.02	0.271	0.263	0.91
		3	0.06	0.298	0.319	0.96	0.00	0.172	0.195	0.94	0.05	0.283	0.296	0.95
		4	0.05	0.309	0.330	0.95	−0.01	0.173	0.188	0.93	0.05	0.309	0.330	0.95

Overall, with the linear settings, Lasso provides a consistent estimator in most cases and largely attenuates the bias compared with SCAD and RF. For more general model settings, RF has potential advantages when the linear model does not hold. The post selection option with RCV variance estimation for BF construction provides consistent estimation on associated parameters with stable CR and is recommended especially when we have sparse high-dimensional data structure.

## 5. Data analysis

We exemplify our methodologies utilizing data from the WHI NPAAS feeding study (*n* = 153), NPAAS biomarker study (*n* = 450), and the comprehensive WHI cohort data [comprising the WHI Observational Study (OS) and the Dietary Modification Trial Control Arm (DM-C), *n* = 122, 970]. A log-transformed self-reported ratio of sodium to potassium intake from FFQ serves as *Q*. Covariates such as age, BMI, race/ethnicity, education level, self-reported physical activity, and smoking status are considered as ***V***. The high-dimensional 24 h urine measurements, acquired via nuclear magnetic resonance (NMR) and gas chromatography-mass spectrometry (GC-MS) platforms, are denoted as ***W***. The disease outcome under consideration is total cardiovascular disease (CVD). The prevalence of CVD events is <10% (41), suggesting that the rare disease assumption is not substantially violated. Follow-up times commence at the moment of FFQ measurement (year-1 visit in DM-C and at enrollment in OS) and persist until the earliest of the specific CVD outcomes under consideration, death, loss to follow-up, or September 30, 2010, whichever occurs first. In our analytical process, hazard rates are modeled as implicitly conditioned on the continued survival of the study subject. This implies that death is not viewed as a source of censoring in our formulation. Rather, death merely constrains the follow-up period during which hazard rate information is collected for the subject. This differs from considering death as censoring non-fatal outcomes, which would be the case in a competing risk formulation.

We scrutinized the normality of the log-transformed self-reported intake (*Q*), the log-transformed metabolites from 24-hour urine measurements (***W***), and the log-transformed evaluated sodium/potassium ratio ($\tilde{X}$) utilizing the NPAAS-FS dataset. No evidence indicated a violation of any normality assumptions (B-H adjusted *p* value > 0.1) (42).

The estimated HR and corresponding 95% confidence interval according to a 20% increase in the sodium-potassium ratio are shown in Table 7 for the methods of Lasso, SCAD, and RF.

**Table 7:** Association between 20% increase in sodium-to-potassium ratio with total CVD in high-dimensional space under Lasso, SCAD, and RF approaches.

**Approach**	**Method**	**Lasso**		**SCAD**		**Random forest**	
		**HR**	**95 CI%**	**HR**	**95 CI%**	**HR**	**95 CI%**
Direct selection with V not fixed	1 2 3 4	1.30	(1.16, 1.46)	1.18	(1.04, 1.34)	1.39	(0.61, 3.18)
		1.09	(1.04, 1.15)	1.05	(0.99, 1.11)	1.10	(0.71, 1.70)
		1.08	(1.00, 1.17)	1.08	(0.97, 1.21)	1.28	(1.00, 1.65)
		1.08	(1.03, 1.13)	1.08	(1.03, 1.13)	1.08	(1.03, 1.13)
Direct-selection with V fixed	1 2 3 4	1.21	(1.04, 1.40)	1.18	(0.80, 1.73)	–	–
		1.06	(1.00, 1.14)	1.05	(1.00, 1.11)	–	–
		1.08	(1.00, 1.18)	1.08	(0.88, 1.33)	–	–
		1.08	(1.03, 1.13)	1.08	(1.03, 1.13)	–	–
Post selection with V not fixed	1 2.3 3 4	1.20	(1.08, 1.33)	1.17	(0.81, 1.69)	1.19	(1.06, 1.34)
		1.08	(1.01, 1.15)	1.07	(0.87, 1.31)	1.08	(1.03, 1.13)
		1.09	(0.98, 1.21)	1.10	(0.97, 1.25)	1.11	(1.05, 1.16)
		1.08	(1.03, 1.13)	1.08	(1.03, 1.13)	1.08	(1.03, 1.13)
Post selection with V fixed	1 2.3 3 4	1.16	(1.03, 1.31)	1.15	(0.87,1.52)	–	–
		1.06	(0.99, 1.14)	1.05	(0.90, 1.22)	–	–
		1.08	(1.00, 1.17)	1.08	(0.97, 1.20)	–	–
		1.08	(1.03, 1.13)	1.08	(1.02, 1.14)	–	–

We observe that the estimated HR is >1 in all cases, indicating a higher risk of CVD with increased sodium-to-potassium ratios, regardless of the different high-dimensional approaches used. These findings are consistent with those reported in previously published studies (20). The most conservative estimate for ${\hat{\tilde{\sigma }}}_{x}^{2}$, 0, is used to construct the BF in Method 2. Moreover, RCV variance estimation is employed to construct the BF for the post selection approach with Method 2. The estimation of the associated parameter derived from Method 2 is smaller in scale compared to Method 1 and is similar to Methods 3 and 4. We note that the 95% CI does not include an HR of 1 with Lasso and RF in most cases, indicating a significant association between calibrated dietary intake and the risk of CVD. Conversely, the 95% CI with SCAD exhibits less efficient results with larger variance, indicating a non-significant association between calibrated dietary intake and the risk of CVD in several instances.

## 6. Discussion

We investigated the prerequisites for a valid biomarker in high-dimensional space for regression calibration purposes. Various methods to handle high-dimensional data (i.e., Lasso, SCAD, and RF) and approaches to variable selection (i.e., direct and post selection) were applied and compared across different scenarios, such as sparsity level and pattern of effect size. This paper offers researchers a comprehensive understanding of how to handle high-dimensional data in calibrated regression studies. Building linear regression models in high-dimensional space presents challenges, such as overfitting to samples and multicollinearity, which can lead to inadequate estimations.

In order to identify the most effective measurements associated with consumed dietary intakes in the feeding study, Lasso, SCAD, and RF were applied for variable selection within the high-dimensional dataset. Overall, Lasso demonstrated more stable results for variable selection compared to the other two approaches. Method 2, with the BF constructed using RCV estimation under the Lasso post selection approach, consistently provided good estimations in most cases.

It is worth noting that various factors, such as filtering conditions and methods for obtaining tuning parameters, can influence the accuracy of the biomarker prediction model when using penalized regression methods and RF. Depending on these choices, the accuracy of the estimated association parameters can vary significantly. Consequently, researchers should carefully consider these factors to achieve the most accurate and reliable results when working with high-dimensional data in calibrated regression studies.

Identifying effective measurements associated with consumed dietary intakes is crucial for biomarker construction. Statistical inference presents challenges with penalized estimators. In this paper, a bootstrapping approach was employed for variance estimation in high-dimensional data for penalized regression and RF. However, there are alternative approaches for variance estimation in high-dimensional data with penalized regression that could be considered in future analyses.

One issue with the estimated covariance matrix relates to zero components. Specifically, when coefficients are zero, the approximate covariance matrix results in zero for estimated variance. Although the estimation of non-zero components is robust, the signs of zero components can be either negative or positive. This issue is also present in the sandwich formula of the covariance matrix developed by (31). Wasserman and Roeder (43) proposed a two-stage procedure for valid inference. Their method involves randomly dividing the data into training and testing datasets. Penalized linear regression is used in the training data to select informative variables in the first stage, while ordinary least squares (OLS) are applied in the testing data to compute standard errors. A drawback of the single-split method is that results may depend on how the data is split. To address this, Meinshausen et al. (44) suggested a multi-split method, which repeats the single-split multiple times. Lockhart et al. (45) introduced the covariance test statistic to test the significance of predictor variables that enter the current Lasso model. For ultra-high-dimensional cases where the sample size is equal to or smaller than the variable dimension, the sure independent screening (SIS) technique proposed by (31) can be considered for variable screening in future work.

## Data availability statement

The data analyzed in this study is subject to the following licenses/restrictions: the data can only be accessed through the collaborative mode as described on the Women's Health Initiative website. Requests to access these datasets should be directed to www.whi.org.

## Author contributions

RD and CZ designed the research and had primary responsibility for the final content. YZ, RD, and CZ derived the methods. YZ performed simulation studies and run the data analysis. RD drafted the paper. YH and RP revised the draft and provide critical feedback to the paper. All authors read and approved the final manuscript.
