# Supplementary material for: Regression calibration utilizing biomarkers developed from high-dimensional metabolites
Source: Front Nutr. 2023 Aug 2;10:1215768. doi: 10.3389/fnut.2023.1215768 (PMC10433218; doi:10.3389/fnut.2023.1215768)
Supplement: Supplementary file 1 [file Data_Sheet_1.PDF]

Supplementary Material: Regression calibration utilizing biomarkers developed from high-dimensional metabolites

Yiwen Zhang, Ran Dai, Ying Huang, Ross L Prentice, and Cheng Zheng

1 ADDITIONAL SIMULATION INFORMATION

1.1 Simulation settings

We simulate data from the following models:

$$
\begin{aligned}
&(Z, V) \sim \mathcal{N}\left(0, \begin{pmatrix} 1-\sigma_x^2 & \rho \\ \rho & 1 \end{pmatrix}\right),\\
&\boldsymbol{W} = b_0 + b_1 X + b_2 V + \epsilon_w,\\
&X = Z + \epsilon_x,\\
&X^* = X + \epsilon_{x^*},\\
&Q = a_0 + a_1 Z + a_2 V + \epsilon_q,\\
&\lambda(t|Z, X, V, \boldsymbol{W}, Q) = \lambda(t|Z, V) = \lambda_0(t)\exp(\theta_z Z + \theta_v V),
\end{aligned}
$$

where Z, V, X and Q are all in one-dimensional space while $\boldsymbol{W}$ is in high-dimensional space. Specifically, we set $\boldsymbol{W}$ in 100-dimensional space. That is, $\boldsymbol{b}_0 = (b_0^1, ..., b_0^{100})^T$, $\boldsymbol{b}_1 = (b_1^1, ..., b_1^{100})^T$ and $\boldsymbol{b}_2 = (b_2^1, ..., b_2^{100})^T$. Here ϵ_x and ϵ_q are independently sampled from normal distributions with mean zero and standard deviations σ_x and σ_q. Censoring time is sampled from a mixture of Unif(0,10) and a point mass at 10 with equal probability and we set $\lambda_0(t) = 0.002t$.

Furthermore, we set $\boldsymbol{b}_2 = (1, .., 1)$, $\sigma_x = 0.2$, $\sigma_{x^*} = 0.5$, $\theta_z = 0.4$, $\theta_v = 0.6$, $\sigma_w = 1$. Then we change the values of $||\boldsymbol{b}_1||_2$, ρ, a_1, a_2 and σ_q to change the range of R^2. All types of R^2 are shown in Table S1 with respect to different patterns, effect sizes and levels of the sparsity of X on $\boldsymbol{W}$ in this chapter. Three representative settings were selected with the sparsity of $\boldsymbol{W}$ equal to 2, 5, and 10. Under each size of sparsity, three different forms on the effect size of $\boldsymbol{W}$ are also generated and compared, including equivalent effect size of X on $\boldsymbol{W}$, and random pattern of effect size on $\boldsymbol{W}$ Below are the settings selected for simulation.
$||\boldsymbol{b}_1||_2 = 1.3$, $\rho = 0.6$, $a_0 = 4$, $a_1 = 1.5$, $\sigma_q = 3$ (setting 1);
$||\boldsymbol{b}_1||_2 = 1.1$, $\rho = 0$, $a_0 = 0.4$, $a_1 = 2$, $\sigma_q = 4$ (setting 2);
$||\boldsymbol{b}_1||_2 = 2$, $\rho = 0.6$, $a_0 = 4$, $a_1 = 1.5$, $\sigma_q = 3$ (setting 3);
where $||\boldsymbol{b}_1||_2$ shown in above settings is the total effect size of X on $\boldsymbol{W}$, that is, $||\boldsymbol{b}_1||_2 = \sqrt{\sum_{i=1}^{100}(b_1^i)^2}$. For example, if we assume the pattern of effect of X on $\boldsymbol{W}$ is equally distributed

with sparse size of 5 using setting 1, then we have:

$$\boldsymbol{b}_1 = (1.1/\sqrt{5}, 1.1/\sqrt{5}, 1.1/\sqrt{5}, 1.1/\sqrt{5}, 1.1/\sqrt{5}, 0..., 0)$$

.

Based on Table S1, almost all types of R^2 are similar to each other for different patterns, sparsity within each setting except for $R^2_{\hat{X}QV}$ and $R^2_{\hat{X}Q|V}$ under the random pattern of effect size. Specifically, the strength of FFQ on long-term dietary intake given personal characteristics in stage 1 is controlled to be relatively low ($R^2_{ZQ|V}$=0.13) in setting 1 and 3 while increased to some extent with $R^2_{ZQ|V}$=0.19 in setting 2. The strength of biomarker on consumed dietary intakes in stage 1 given personal characteristics generally follow an increasing trend from setting 1 to setting 3 with $R^2_{ZW|V}$ from 0.49 to 0.68.

1.2 Additional Simulation Results when not forcing personal characteristics in the model

In tables S2S3, we present the simulation results when not forcing personal characteristics in the model for direct and post-selection corrected biomarkers with the Lasso penalty.

In tables , we present the simulation results when not forcing personal characteristics in the model for direct and post-selection corrected biomarkers with the SCAD penalty.

In tables , we present the simulation results when not forcing personal characteristics in the model for RF based biomarkers.

Table S1. List of R^2 for the three settings under different patterns and sparsity

Pattern	Type of R^2	Setting 1			Setting 2			Setting 3		
		S2	S5	S10	S2	S5	S10	S2	S5	S10
Same	R^2_{ZWV}	0.68	0.68	0.67	0.52	0.51	0.50	0.80	0.79	0.79
	$R^2_{ZW\|V}$	0.49	0.48	0.47	0.52	0.51	0.50	0.67	0.67	0.66
	R^2_{ZQV}	0.46	0.46	0.46	0.19	0.19	0.19	0.46	0.46	0.46
	$R^2_{ZQ\|V}$	0.13	0.13	0.13	0.19	0.19	0.19	0.13	0.13	0.13
	R^2_{ZWQV}	0.70	0.70	0.70	0.57	0.56	0.56	0.81	0.80	0.80
	$R^2_{ZWQ\|V}$	0.52	0.51	0.51	0.57	0.56	0.56	0.69	0.68	0.68
	$R^2_{X^*WV}$	0.56	0.55	0.55	0.44	0.43	0.42	0.66	0.66	0.65
	$R^2_{X^*W\|V}$	0.37	0.37	0.36	0.44	0.43	0.42	0.52	0.51	0.50
	$R^2_{X^*WQV}$	0.57	0.57	0.56	0.47	0.46	0.46	0.66	0.66	0.66
	$R^2_{X^*WQ\|V}$	0.40	0.39	0.38	0.47	0.46	0.46	0.52	0.52	0.51
	$R^2_{\hat{X}QV}$	0.50	0.51	0.52	0.03	0.07	0.07	0.46	0.47	0.48
	$R^2_{\hat{X}Q\|V}$	0.05	0.06	0.06	0.03	0.07	0.07	0.07	0.08	0.08
Random	R^2_{ZWV}	0.68	0.68	0.69	0.52	0.53	0.53	0.80	0.80	0.80
	$R^2_{ZW\|V}$	0.49	0.49	0.49	0.52	0.53	0.53	0.67	0.68	0.68
	R^2_{ZQV}	0.46	0.46	0.46	0.19	0.19	0.19	0.46	0.46	0.46
	$R^2_{ZQ\|V}$	0.13	0.13	0.13	0.19	0.19	0.19	0.13	0.13	0.13
	R^2_{ZWQV}	0.71	0.71	0.71	0.57	0.58	0.58	0.81	0.81	0.81
	$R^2_{ZWQ\|V}$	0.52	0.53	0.53	0.57	0.58	0.58	0.69	0.69	0.69
	$R^2_{X^*WV}$	0.56	0.56	0.56	0.44	0.44	0.44	0.66	0.66	0.66
	$R^2_{X^*W\|V}$	0.37	0.37	0.37	0.44	0.44	0.44	0.52	0.52	0.52
	$R^2_{X^*WQV}$	0.57	0.57	0.57	0.47	0.47	0.47	0.66	0.66	0.66
	$R^2_{X^*WQ\|V}$	0.40	0.40	0.40	0.47	0.47	0.47	0.53	0.52	0.53
	$R^2_{\hat{X}QV}$	0.48	0.29	0.23	0.00	0.01	0.02	0.43	0.14	0.07
	$R^2_{\hat{X}Q\|V}$	0.04	0.00	0.00	0.00	0.01	0.02	0.05	0.00	0.01

Table S2. Simulation results with direct-Lasso selection not forcing personal characteristics in the model

			Setting 1				Setting 2				Setting 3			
Pattern	Sparsity	Method	Bias	SD	SE	CR	Bias	SD	SE	CR	Bias	SD	SE	CR
Same	2	1	0.65	0.730	0.814	0.89	0.52	0.459	0.455	0.82	0.26	0.437	0.454	0.94
		2	0.03	0.301	0.339	0.96	0.01	0.192	0.206	0.95	0.02	0.278	0.287	0.97
		3	-0.03	0.238	0.265	0.97	-0.02	0.157	0.184	0.97	-0.02	0.244	0.269	0.95
		4	-0.01	0.280	0.355	0.94	-0.02	0.158	0.194	0.95	-0.01	0.280	0.355	0.94
	5	1	1.14	3.476	1.526	0.93	0.69	0.945	0.596	0.86	0.32	0.558	0.518	0.95
		2	0.03	0.524	0.515	0.95	-0.01	0.222	0.215	0.97	0.01	0.286	0.299	0.95
		3	-0.04	0.243	0.275	0.96	-0.02	0.163	0.187	0.97	-0.02	0.264	0.277	0.95
		4	-0.01	0.280	0.355	0.94	-0.02	0.158	0.194	0.95	-0.01	0.280	0.355	0.94
	10	1	1.16	2.053	6.957	0.94	0.98	2.178	0.909	0.92	0.36	0.605	1.228	0.94
		2	-0.07	0.253	1.764	0.96	-0.08	0.165	0.233	0.98	-0.03	0.247	0.302	0.95
		3	-0.04	0.243	0.281	0.95	-0.03	0.156	0.193	0.96	-0.03	0.259	0.287	0.95
		4	-0.01	0.280	0.355	0.94	-0.02	0.158	0.194	0.95	-0.01	0.280	0.355	0.94
Random	2	1	0.54	0.621	0.713	0.91	0.51	0.422	0.456	0.83	0.21	0.378	0.421	0.94
		2	-0.01	0.277	0.299	0.97	0.03	0.208	0.214	0.97	0.00	0.256	0.275	0.95
		3	-0.06	0.213	0.254	0.96	-0.01	0.178	0.182	0.97	-0.05	0.222	0.252	0.96
		4	-0.01	0.258	0.324	0.97	0.00	0.177	0.191	0.96	-0.01	0.258	0.324	0.97
	5	1	0.62	0.657	0.829	0.91	0.58	0.461	0.503	0.88	0.25	0.369	0.458	0.95
		2	-0.01	0.241	0.336	0.99	0.05	0.193	0.222	0.95	0.01	0.235	0.294	1.00
		3	-0.04	0.203	0.265	0.96	0.00	0.150	0.193	0.96	-0.03	0.202	0.264	0.97
		4	0.01	0.250	0.323	0.97	0.01	0.161	0.206	0.98	0.01	0.250	0.323	0.97
	10	1	0.88	1.015	0.878	0.86	0.61	0.518	0.513	0.82	0.34	0.499	0.458	0.88
		2	0.03	0.313	0.325	0.94	0.03	0.203	0.215	0.95	0.05	0.294	0.281	0.90
		3	-0.01	0.240	0.254	0.93	-0.01	0.172	0.182	0.93	0.00	0.245	0.252	0.94
		4	0.05	0.309	0.330	0.95	-0.01	0.173	0.188	0.93	0.05	0.309	0.330	0.95

Table S3. Simulation results with post-Lasso selection not forcing personal characteristics in the model

			Setting 1				Setting 2				Setting 3			
Pattern	Sparsity	Method	Bias	SD	SE	CR	Bias	SD	SE	CR	Bias	SD	SE	CR
Same	2	1	0.42	0.597	0.672	0.94	0.34	0.346	0.366	0.90	0.16	0.396	0.378	0.93
		2.1	-0.22	0.200	0.227	0.72	-0.11	0.145	0.164	0.91	-0.11	0.202	0.213	0.89
		2.2	0.15	0.394	0.446	0.95	0.06	0.208	0.230	0.93	0.06	0.324	0.314	0.94
		2.3	0.00	0.305	0.334	0.94	-0.02	0.186	0.194	0.94	-0.01	0.280	0.267	0.93
		3	-0.02	0.252	0.303	0.97	-0.01	0.168	0.187	0.97	-0.02	0.256	0.286	0.95
		4	-0.01	0.280	0.355	0.94	-0.02	0.158	0.194	0.95	-0.01	0.280	0.355	0.94
	5	1	0.38	0.941	0.800	0.95	0.47	0.676	0.454	0.88	0.18	0.495	0.408	0.94
		2.1	-0.26	0.183	0.244	0.78	-0.17	0.144	0.172	0.78	-0.12	0.206	0.218	0.88
		2.2	0.09	0.522	0.493	0.97	0.11	0.349	0.266	0.93	0.06	0.364	0.327	0.95
		2.3	-0.08	0.320	0.335	0.95	-0.06	0.216	0.205	0.93	-0.03	0.306	0.268	0.93
		3	-0.02	0.296	0.326	0.95	-0.02	0.182	0.189	0.98	0.00	0.418	0.294	0.97
		4	-0.01	0.280	0.355	0.94	-0.02	0.158	0.194	0.95	-0.01	0.280	0.355	0.94
	10	1	0.59	1.237	1.937	0.97	0.72	1.267	0.731	0.88	0.27	0.580	0.510	0.92
		2.1	-0.31	0.363	0.317	0.75	-0.29	0.268	0.217	0.68	-0.17	0.175	0.226	0.89
		2.2	0.15	0.566	1.066	0.98	0.20	0.546	0.388	0.93	0.10	0.384	0.382	0.94
		2.3	-0.08	0.343	0.748	0.93	-0.09	0.351	0.247	0.94	-0.04	0.275	0.284	0.91
		3	-0.01	0.284	0.357	0.95	-0.02	0.169	0.200	0.96	-0.01	0.269	0.381	0.94
		4	-0.01	0.280	0.355	0.94	-0.02	0.158	0.194	0.95	-0.01	0.280	0.355	0.94
Random	2	1	0.41	0.568	0.645	0.93	0.36	0.366	0.370	0.86	0.14	0.336	0.379	0.96
		2.1	-0.15	0.212	0.224	0.79	-0.11	0.166	0.166	0.8	-0.09	0.208	0.217	0.94
		2.2	0.11	0.372	0.395	0.94	0.09	0.240	0.240	0.94	0.04	0.276	0.307	0.95
		2.3	-0.01	0.298	0.312	0.94	0.00	0.209	0.203	0.95	-0.02	0.245	0.267	0.95
		3	-0.01	0.298	0.300	0.96	0.00	0.186	0.188	0.97	-0.01	0.278	0.280	0.96
		4	-0.01	0.258	0.324	0.97	0.00	0.177	0.191	0.96	-0.01	0.258	0.324	0.97
	5	1	0.43	0.522	0.694	0.94	0.37	0.349	0.409	0.88	0.16	0.323	0.408	0.96
		2.1	-0.18	0.199	0.255	0.85	-0.13	0.139	0.171	0.88	-0.09	0.185	0.236	0.95
		2.2	0.11	0.311	0.431	0.95	0.10	0.219	0.266	0.96	0.06	0.261	0.329	0.98
		2.3	-0.03	0.239	0.337	0.99	-0.01	0.178	0.213	0.96	-0.01	0.230	0.285	0.98
		3	-0.01	0.234	0.547	0.94	0.00	0.156	0.199	0.96	-0.01	0.230	0.312	0.95
		4	0.01	0.250	0.323	0.97	0.01	0.161	0.206	0.98	0.01	0.250	0.323	0.97
	10	1	0.59	0.738	0.683	0.89	0.38	0.396	0.392	0.88	0.24	0.433	0.399	0.87
		2.1	-0.19	0.203	0.242	0.79	-0.16	0.161	0.166	0.75	-0.07	0.223	0.227	0.89
		2.2	0.18	0.415	0.405	0.92	0.10	0.242	0.253	0.92	0.11	0.335	0.315	0.91
		2.3	0.02	0.316	0.313	0.93	-0.02	0.187	0.202	0.92	0.04	0.286	0.272	0.92
		3	0.04	0.298	0.320	0.95	0.00	0.182	0.189	0.95	0.04	0.297	0.293	0.95
		4	0.05	0.309	0.330	0.95	-0.01	0.173	0.188	0.93	0.05	0.309	0.330	0.95

Table S4. Simulation results for direct-SCAD selection not forcing personal characteristics in the model

			Setting 1				Setting 2				Setting 3			
Pattern	Sparsity	Method	Bias	SD	SE	CR	Bias	SD	SE	CR	Bias	SD	SE	CR
Same	2	1	0.52	0.941	0.643	0.91	0.45	0.578	0.420	0.84	0.19	0.379	0.408	0.95
		2	-0.04	0.237	0.283	0.95	-0.05	0.195	0.184	0.95	-0.03	0.240	0.262	0.93
		3	0.00	0.275	0.300	0.96	-0.01	0.179	0.188	0.97	0.01	0.262	0.307	0.96
		4	-0.01	0.280	0.355	0.94	-0.02	0.158	0.194	0.95	-0.01	0.280	0.355	0.94
	5	1	0.73	1.848	1.324	0.87	0.91	1.238	1.124	0.89	0.37	1.066	0.566	0.97
		2	-0.10	0.322	0.405	0.92	-0.10	0.226	0.210	0.91	-0.04	0.345	0.277	0.92
		3	-0.02	0.282	0.317	0.94	-0.03	0.157	0.195	0.97	0.01	0.329	0.330	0.95
		4	-0.01	0.280	0.355	0.94	-0.02	0.158	0.194	0.95	-0.01	0.280	0.355	0.94
	10	1	1.75	2.790	3.470	0.90	2.78	4.566	4.244	0.87	0.56	0.959	0.940	0.93
		2	-0.14	0.636	0.684	0.92	-0.12	0.343	0.462	0.86	-0.08	0.268	0.316	0.92
		3	-0.05	0.246	0.293	0.94	-0.02	0.164	0.202	0.96	-0.02	0.263	0.337	0.96
		4	-0.01	0.280	0.355	0.94	-0.02	0.158	0.194	0.95	-0.01	0.280	0.355	0.94
Random	2	1	0.42	0.555	0.629	0.90	0.42	0.392	0.408	0.88	0.15	0.349	0.380	0.95
		2	-0.05	0.244	0.270	0.95	-0.01	0.189	0.193	0.95	-0.04	0.233	0.248	0.94
		3	-0.04	0.223	0.274	0.96	0.00	0.189	0.184	0.96	-0.04	0.231	0.267	0.95
		4	-0.01	0.258	0.324	0.97	0.00	0.177	0.191	0.96	-0.01	0.258	0.324	0.97
	5	1	0.54	0.670	0.755	0.92	0.46	0.369	0.453	0.91	0.19	0.331	0.413	0.98
		2	-0.06	0.221	0.293	0.93	-0.01	0.169	0.197	0.96	-0.02	0.253	0.265	0.98
		3	-0.04	0.210	0.281	0.95	0.00	0.152	0.197	0.96	-0.03	0.217	0.283	0.94
		4	0.01	0.250	0.323	0.97	0.01	0.161	0.206	0.98	0.01	0.250	0.323	0.97
	10	1	0.70	0.874	0.936	0.87	0.53	0.564	0.496	0.81	0.28	0.464	0.427	0.88
		2	-0.04	0.273	0.347	0.89	-0.05	0.173	0.188	0.94	0.00	0.265	0.249	0.90
		3	0.01	0.281	0.270	0.94	-0.01	0.172	0.185	0.93	0.01	0.258	0.269	0.94
		4	0.05	0.309	0.330	0.95	-0.01	0.173	0.188	0.93	0.05	0.309	0.330	0.95

Table S5. Simulation results for post-SCAD selection not forcing personal characteristics in the model

			Setting 1				Setting 2				Setting 3			
Pattern	Sparsity	Method	Bias	SD	SE	CR	Bias	SD	SE	CR	Bias	SD	SE	CR
Same	2	1	0.76	3.202	2.103	0.92	0.47	0.576	0.526	0.86	0.17	0.405	0.439	0.92
		2.1	-0.25	0.187	0.421	0.75	-0.06	0.339	0.284	0.91	-0.12	0.203	0.226	0.86
		2.2	0.18	0.590	0.446	0.93	0.10	0.277	0.246	0.93	0.06	0.304	0.312	0.95
		2.3	0.09	0.605	0.602	0.94	0.01	0.287	0.276	0.94	0.00	0.306	0.325	0.93
		3	-0.02	0.260	0.293	0.94	0.01	0.301	0.193	0.97	-0.02	0.247	0.292	0.96
		4	-0.01	0.280	0.355	0.94	-0.02	0.158	0.194	0.95	-0.01	0.280	0.355	0.94
	5	1	0.80	1.438	2.574	0.91	0.82	1.209	1.697	0.93	0.26	0.521	0.657	0.94
		2.1	-0.27	0.290	0.279	0.74	-0.06	0.493	0.339	0.90	-0.12	0.276	0.269	0.88
		2.2	-0.30	3.900	0.527	0.94	0.14	0.430	0.486	0.93	0.07	0.419	0.363	0.95
		2.3	-0.10	0.782	2.144	0.93	-0.07	2.098	0.720	0.92	-0.01	0.332	0.470	0.95
		3	-0.02	0.346	0.327	0.96	-0.02	0.191	0.193	0.97	-0.01	0.380	0.309	0.97
		4	-0.01	0.280	0.355	0.94	-0.02	0.158	0.194	0.95	-0.01	0.280	0.355	0.94
	10	1	1.14	2.148	16.145	0.96	1.27	2.461	6.402	0.91	0.42	0.774	1.518	0.92
		2.1	-0.41	0.532	0.622	0.80	0.15	4.712	0.523	0.88	-0.19	0.214	0.303	0.88
		2.2	0.32	1.334	1.225	0.96	0.20	0.661	0.381	0.95	0.10	0.441	0.402	0.94
		2.3	-0.13	0.842	10.394	0.96	-0.19	2.013	1.073	0.93	0.00	0.493	0.646	0.92
		3	-0.03	0.282	0.340	0.95	-0.02	0.174	0.203	0.96	-0.02	0.272	0.304	0.95
		4	-0.01	0.280	0.355	0.94	-0.02	0.158	0.194	0.95	-0.01	0.280	0.355	0.94
Random	2	1	-0.16	5.530	3.078	0.94	0.43	0.741	0.543	0.87	0.16	0.411	0.444	0.95
		2.1	-0.17	0.320	1.417	0.82	-0.13	0.190	0.220	0.85	-0.11	0.211	0.288	0.92
		2.2	0.13	0.370	0.432	0.94	0.11	0.255	0.254	0.94	0.06	0.305	0.318	0.92
		2.3	0.03	0.444	0.690	0.95	0.03	0.254	0.291	0.93	-0.01	0.275	0.337	0.95
		3	2.55	25.860	0.321	0.96	0.01	0.212	0.192	0.96	-0.08	0.569	0.298	0.96
		4	-0.01	0.258	0.324	0.97	0.00	0.177	0.191	0.96	-0.01	0.258	0.324	0.97
	5	1	0.52	0.641	2.078	0.93	0.46	0.500	0.612	0.92	0.19	0.372	0.459	0.95
		2.1	-0.27	0.213	0.319	0.83	-0.13	0.285	0.213	0.82	-0.13	0.186	0.254	0.90
		2.2	0.14	0.337	0.489	0.96	0.10	0.206	0.274	0.97	0.06	0.261	0.348	1.00
		2.3	0.04	0.483	0.735	0.96	0.01	0.225	0.377	0.98	0.00	0.229	0.394	0.99
		3	-0.02	0.226	0.362	0.96	0.00	0.160	0.203	0.96	-0.01	0.225	0.308	0.97
		4	0.01	0.250	0.323	0.97	0.01	0.161	0.206	0.98	0.01	0.250	0.323	0.97
	10	1	1.27	3.524	5.354	0.90	0.25	3.285	1.265	0.94	0.32	0.559	0.598	0.93
		2.1	-0.22	0.326	0.422	0.68	-0.18	0.200	0.204	0.79	-0.09	0.245	0.225	0.81
		2.2	0.23	0.513	0.483	0.93	0.11	0.260	0.267	0.92	0.11	0.333	0.327	0.92
		2.3	0.16	1.085	1.196	0.91	-0.11	1.113	1.551	0.95	0.01	0.288	0.318	0.92
		3	0.02	0.273	0.325	0.94	0.00	0.183	0.192	0.95	0.02	0.267	0.296	0.95
		4	0.05	0.309	0.330	0.95	-0.01	0.173	0.188	0.93	0.05	0.309	0.330	0.95
